# Restraining hospitalized children for procedures: practices of nursing professionals in Brazil*

**DOI:** 10.1590/1980-220X-REEUSP-2025-0453en

**Published:** 2026-05-29

**Authors:** Ronaldo Antonio da Silva, Maria Aparecida Munhoz Gaíva

**Affiliations:** 1 Universidade do Estado de Mato Grosso, Diamantino, MT, Brazil.; 2 Universidade Federal de Mato Grosso, Cuiabá, MT, Brazil.

**Keywords:** Child, Hospitalized, Critical Pathways, Restraint, Physical, Nursing, Team, Nursing Care

## Abstract

**Objective:**

To describe the restraint practices used by Brazilian nursing professionals when performing healthcare procedures on hospitalized children.

**Method:**

A cross-sectional study was conducted with 897 professionals working in the care of hospitalized children in the country. Data were collected between May 2024 and January 2025, using a questionnaire with sociodemographic information and another about restraint practices, which underwent content validation and pre-testing. The bank was analyzed in software R.

**Results:**

Restraint was characterized as a common method; however, professionals reported that they did not receive training during their education and/or professional practice to perform it. The most commonly used techniques were physical, with support, and psychological, in procedures such as venipuncture, medication administration, and catheterization. The decision to use it was related to the child’s behavior, ensuring their safety, and their refusal to cooperate.

**Conclusion:**

The restraint practices used by Brazilian nursing staff for pediatric care were described based on techniques, reasons, consent, family participation, control of potential harm, and documentation in medical records.

## INTRODUCTION

Restraint is defined as any method that limits a person’s movement, physical activity, or normal access to their body, and has been frequently adopted by healthcare professionals in pediatric care^(1,2,3)^. This practice is used during a child’s hospitalization for varied health procedures, such as: venipuncture, medication administration, catheterization, blood tests, and X-rays^(1,2,3)^. However, child restraint is surrounded by uncertainties and controversies regarding the ethical and moral implications related to professional practice, as well as the short-, medium-, and long-term risks and consequences for the lives and health of the children who are restrained^(3,4)^.

It is known that the practice of restraint during healthcare procedures can have harmful effects on the child, as scientific evidence indicates. Swedish researchers, analyzing the narratives of children undergoing various non-urgent healthcare procedures, highlighted that, for these children, restraint meant emotional distress^(4)^. In this research, the children reported feeling hurt when forced to undergo such procedures, and that the memories of these moments remained for a long time. Furthermore, according to the authors’ inference, these negative memories may affect the child’s interaction with professionals in future healthcare settings.

Within this context, it is known that anticipatory anxiety is a reality in the lives of hospitalized children undergoing health procedures. According to the literature, they may experience distress both before the procedures and when experiencing restraint against their will^(1,4,5,6)^. Among the factors that trigger and intensify feelings of anticipatory anxiety before a health procedure is the negative experience of children and their families with previous procedures and hospitalizations^(7)^.

Regarding the practical application of restraint, a recent scoping review underlined that, among healthcare professionals, those comprising the nursing team were cited in 61.5% of studies as being involved in child restraint^(3)^. Additionally, studies conducted in different countries were cataloged, with particular emphasis on those from the European continent. However, no published studies on this subject were identified in Brazil, indicating a gap in the country’s scientific literature^(3)^. Given the problem presented, the following research question was raised: what restraint practices are used by Brazilian nursing professionals when performing healthcare procedures on hospitalized children?

This research has the potential to provide insight into the daily practices of nursing professionals in healthcare services in Brazil and, thus, understand how the phenomenon of restraint of hospitalized children manifests itself in this social and cultural context, for which there is still little data available. These results will contribute to the international scientific literature, broadening the scope to support approaches that prioritize guaranteeing rights and ensuring children’s physical, emotional, and psychological well-being during non-urgent health procedures. Therefore, this study had as objective to describe the restraint practices used by Brazilian nursing professionals when performing healthcare procedures on hospitalized children.

## METHOD

### Design of Study

This is a cross-sectional study.

### Population, Selection Criteria, and Sample

The study took place in Brazil, a country divided into five macro-regions for political and administrative organization purposes: North, Northeast, South, Southeast, and Central-West, encompassing the 27 Federative Units^(8)^.

The population consisted of nurses, nursing technicians and/or assistants who worked in the care of hospitalized children in the five macro-regions of the country. Sampling was conducted through self-selection, a non-probabilistic technique employed when participants have characteristics relevant to the research and volunteer to participate^(9)^. In this study, the inclusion criterion was having at least six months of professional experience in pediatric and/or neonatal nursing care, a period considered adequate to ensure familiarity and experience with the practices of restraining hospitalized children. Professionals who participated in the pre-test were excluded.

Regarding the sample size, when using data from the survey on the profile of nursing in Brazil^(10)^, proportional sampling calculations were performed for each Brazilian macro-region in the strata of nurses and nursing technicians and assistants. With a 95% confidence level, a minimum sample of 385 subjects was required for each stratum. The sample consisted of 897 nursing professionals.

### Data Collection

Data collection took place between May 2024 and January 2025. The following were used: a questionnaire with questions regarding sociodemographic information and another about the experience with restraint in healthcare procedures for children during hospitalization. This second one was developed based on a scoping review of the topic^(3)^ and is subject to content validation to achieve legitimacy and credibility. Six specialists participated in this stage, including three professors and researchers, a pediatric supervising nurse, a pediatric assistant nurse, and an assistant nurse in the Neonatal Intensive Care Unit (NICU). After review of the domains and items, a Content Validity Index (CVI) of 0.86 was presented, which was considered a satisfactory level of agreement^(11,12)^.

Thus, variables related to sociodemographic characteristics (professional category, age, sex, marital status, presence of children, and macro-region of residence); professional training (having studied or recently completed a course on restraint, having or pursuing postgraduate studies in the pediatric/neonatological area, and the modality); professional activity (area and length of work); and restraint practices (use, who performs it, technique, associated procedure, reasons, perception of need by age/sex, communication/consent with family member and child, presence/help of family member, harm control, and record keeping in medical records, including the type of information recorded) were investigated.

Following this process, the questionnaires were registered in *Google Forms* ^®^ and submitted to pre-testing, being applied to nine nursing professionals who worked in an emergency care unit providing urgent and emergency care to newborns, neonates and/or children in the state of Mato Grosso, Central-West macro-region. Later, the link for the questionnaires and the Free Informed Consent Form were sent to the participants by *e-mail*, social media (*Instagram* ^®^ and/or *Facebook* ^®^), *WhatsApp* ^®^. Furthermore, the link was shared across the country’s five macro-regions with the support of professional associations, educational institutions, research groups, and hospitals providing pediatric care.

### Data Analysis and Treatment

The database was generated in a spreadsheet in Microsoft Excel^®^, which underwent review by researchers to ensure consistency and integrity. Additionally, the columns were renamed to facilitate processing in the analysis software. After that, the database was analyzed using the R software^(13)^, with calculation of absolute and relative frequencies for qualitative and quantitative variables, measures of central tendency and dispersion, including mean, standard deviation, minimum and maximum values.

### Ethical Aspects

This study followed the ethical precepts of research with human beings in Brazil and was approved by the Research Ethics Committee of the Universidade Federal de Mato Grosso (CEP-SAÚDE/UFMT) under opinion number 6.672.454/2024 and CAAE number 76648023.4.0000.8124, in accordance with resolutions number 466/2012 and 510/2016 of the National Health Council^(14,15)^. It followed the guidelines established in Circular Letter No. 2/2021 from the National Research Ethics Committee (CONEP), which guides the procedures for research with any stage in a virtual environment in the country^(16)^.

## RESULTS

This study included 897 nursing professionals, whose sociodemographic, educational, and professional characteristics are presented in Table 1. The largest proportion of professionals resided in the Southeast macro-region (n = 558; 62.21%), followed by the Central-West (n = 113; 12.60%), South (n = 91; 10.14%), Northeast (n = 68; 7.58%) and North (n = 67; 7.47%). Regarding professional category, the majority were nurses (n = 462; 51.51%), followed by nursing technicians (n = 349; 38.91%) and nursing assistants (n = 86; 9.59%). The participants’ ages ranged from 18 to 72 years, with a mean of 40.9 years and a standard deviation of 10.12 years, indicating a diverse age range. The vast majority of respondents were female (n = 785; 87.51%), and in terms of marital status, most declared themselves married (n = 472; 52.62%), and regarding the presence of children, 627 professionals (69.90%) stated that they had children.

**Table 1:** Sociodemographic, educational, and professional characteristics of the nursing professionals studied – Cuiabá, MT, Brazil, 2025.

Variables	n (%)	Mean (SD)
Professional category		
Nurse	462 (51.51%)	
Nursing technician	349 (38.91%)	
Nursing assistant	86 (9.59%)	
Age (years)		40.87 (10.12)
Sex		
Male	112 (12.49%)	
Female	785 (87.51%)	
Marital status		
Single	326 (36.34%)	
Married	472 (52.62%)	
Divorced	90 (10.03%)	
Widow(er)	9 (1.0%)	
Had child(ren)	
Yes	627 (69.90%)	
No	270 (30.10%)	
Macro-region of residence	
North	67 (7.47%)	
North East	68 (7.58%)	
Central-West	113 (12.60%)	
Southeast	558 (62.21%)	
South	91 (10.14%)	
Studied content about restraint during training		
Yes	285 (31.77%)	
No	612 (68.23%)	
Took training or refresher course on restraint in the last 12 months		
Yes	81 (9.03%)	
No	816 (90.97%)	
Had a graduate degree in the pediatric/neonatological field		
Yes	227 (25.31%)	
No	670 (74.69%)	
Graduate program modality in the area		
Residence	22 (9.69%)	
Graduate certificate course	150 (66.08%)	
Professional Master's Degree	9 (3.96%)	
Academic Master's Degree	19 (8.37%)	
Doctorate degree	20 (8.81%)	
Postdoctoral degree	7 (3.08%)	
Was doing graduate studies in the field.		
Yes	67 (7.47%)	
No	830 (92.53%)	
Graduate course modality in progress		
Residence	10 (14.93%)	
Graduate certificate course	35 (52.24%)	
Professional Master's Degree	1 (1.49%)	
Academic Master's Degree	6 (8.96%)	
Doctorate degree	14 (20.90%)	
Postdoctoral degree	1 (1.49%)	
Professional field of activity^*^		
Newborn health	408 (45.48%)	
Child health	588 (65.55%)	
Adolescent health	491 (54.74%)	
Length of employment (years)		7.2 (8.69)

*The participant could select more than one option; SD: Standard deviation.

In respect of training, 612 (68.23%) professionals reported that they had not been offered any content related to child restraint during their undergraduate or technical/auxiliary nursing course. With regard to continuing education, 816 (90.97%) stated that they had not participated in any training or refresher course on child restraint in the last 12 months. Concerning education, 227 professionals (25.31%) indicated having some type of postgraduate degree in the pediatric or neonatological area, with specialization being the most frequent type (n = 150; 66.08%), followed by residency (n = 22; 9.69%), doctorate (n = 20; 8.81%), academic master’s degree (n = 19; 8.37%), professional master’s degree (n = 9; 3.96%) and postdoctoral studies (n = 7; 3.08%). At the time of data collection, 67 professionals (7.47%) were pursuing some form of postgraduate study in these areas; specialization was again the main type of program (n = 35; 52.24%), followed by doctorate degree (n = 14; 20.9%) and residency (n = 10; 14.93%).

Regarding the professional activity area, participants were able to indicate multiple areas, with the majority working in child health (n = 588; 65.55%), followed by those working in adolescent health (n = 491; 54.74%) and newborn health (n = 408; 45.48%). With reference to the length of time working in pediatric and/or neonatal nursing, data showed a variation between 1 and 50 years of experience (7.2 ± 8.69).

Table 2 highlights the restraint practices used by Brazilian nursing professionals when performing healthcare procedures on hospitalized children. The majority of nursing professionals (n = 760; 80.73%) reported having used the restraint method to perform health procedures on hospitalized children, demonstrating a widespread adoption of this practice in the hospital setting. It was also found that nursing technicians (n = 647; 72.13%) were reported as those who most frequently performed restraint in the workplace.

**Table 2:** Restraint practices used by Brazilian nursing professionals when performing healthcare procedures on hospitalized children – Cuiabá, MT, Brazil, 2025.

Variables	n (%)
Used restraint	
Yes	760 (84.73%)
No	137 (15.27%)
Nursing professional who performs restraint most frequently in their workplace	
Nurse	175 (19.51%)
Nursing technician	647 (72.13%)
Nursing assistant	75 (8.36%)
Technique used in their service^*^	
Physical	
Yes	590 (65.77%)
No	307 (34.23%)
Mechanical	
Yes	257 (28.65%)
No	640 (71.35%)
Chemical	
Yes	202 (22.52%)
No	695 (77.48%)
Psychological	
Yes	349 (38.91%)
No	548 (61.09%)
Supported restraint	
Yes	441 (49.16%)
No	456 (50.84%)
Health procedure^*^	
Medication administration	
Yes	448 (49.94%)
No	449 (50.06%)
Dressing	
Yes	272 (30,32%)
No	625 (69,68%)
Immunization	
Yes	255 (28.43%)
No	642 (71.57%)
Venipuncture	
Yes	795 (88.63%)
No	102 (11.37%)
Catheterization	
No	384 (42.81%)
Yes	513 (57.19%)
Reason(s) for using restraint^*^	
Age	
Yes	343 (38.24%)
No	554 (61.76%)
Behavior (agitated/aggressive)	
Yes	731 (81.49%)
No	166 (18.51%)
Refusal to cooperate	
Yes	476 (53.08%)
No	421 (46.93%)
Presence of any disability	
Yes	228 (25.42%)
No	669 (74.58%)
Comprehension ability	
Yes	429 (47.83%)
No	468 (52.17%)
Ensurance of the child’s safety	
Yes	720 (80.27%)
No	177 (19.73%)
Need to complete the procedure	
Yes	460 (51.28%)
No	437 (48.72%)
Limited time to complete the procedure	
Yes	192 (21.40%)
No	705 (78.60%)
Age-based restraint	
Yes	803 (89.52%)
No	94 (10.48%)
Sex restraint	
Yes	268 (29.88%)
No	629 (70.12%)
Informed the family member and/or guardian	
Yes	814 (90.75%)
No	31 (3.46%)
Sometimes	52 (5.8%)
Informed the child	
Yes	568 (63.32%)
No	105 (11.71%)
Sometimes	224 (24.97%)
Requested consent from a family member and/or guardian	
Yes	617 (68.78%)
No	191 (21.29%)
Sometimes	89 (9.92%)
Asked for the child’s consent	
Yes	304 (33.89%)
No	398 (44.37%)
Sometimes	195 (21.74%)
The family member remains present during the restraint procedure
Yes	757 (84.39%)
No	24 (2.68%)
Sometimes	116 (12.93%)
The family member is instructed to help with the physical restraint of the child	
Yes	648 (76.25%)
No	64 (7.13%)
Sometimes	149 (16.61%)
Control of potential damage	
Yes	464 (51.73%)
No	227 (25.31%)
Sometimes	206 (22.97%)
Control strategies^*^	
Family member’s lap	
Yes	392 (43.70%)
No	505 (56.30%)
Ointment to prevent bruising	
Yes	51 (5.69%)
No	846 (94.31%)
Positive reinforcement (certificates and gifts)	
Yes	175 (19.51%)
No	722 (80.49%)
Others	
Yes	56 (6.24%)
No	841 (93.76%)
Recorded in the medical record	
Yes	630 (70.23%)
No	161 (17.95%)
Sometimes	106 (11.82%)
Information recorded^*^	
Procedure and what required the use of restraint	
Yes	494 (55.07%)
No	403 (44.93%)
Type of restraint used	
Yes	248 (27.65%)
No	649 (72.35%)
Healthcare professional who helped restrain the child	
Yes	153 (17.06%)
No	744 (82.94%)
Presence of family member/guardian	
Yes	262 (29,21%)
No	635 (70.79%)
Consent of family member/guardian	
Yes	262 (29,21%)
No	635 (70.79%)
Child’s consent	
Yes	87 (9.70%)
No	810 (90.30%)
Aspects contributing to the decision to restrain	
Yes	185 (20.62%)
No	712 (79.38%)

*The participant could select more than one option.

Regarding the restraint technique used by professionals, physical restraint was the most frequent (n = 590; 65.77%), followed by restraint with support (n = 441; 49.16%) and psychological restraint (n = 349; 38.91%). In the matter of healthcare procedures in which restraint was most frequently used by nursing professionals, the following stood out: venipuncture (n = 795; 88.63%), medication administration (n = 448; 49.94%), and catheterization (n = 384; 42.81%).

The reasons mentioned by nursing professionals for using restraint on hospitalized children were: the child’s behavior (n = 731; 81.49%), ensuring their safety (n = 720; 80.27%), and also their refusal to cooperate (n = 476; 53.08%). Regarding the need for restraint, the majority of professionals (n = 629; 70.12%) did not identify a difference based on the children’s sex.

When analyzing the data on the explanation of the type of restraint adopted, it was observed that the largest proportion of nursing professionals considered informing the family and/or the guardian (n = 814; 90.75%) rather than the child (n = 568; 63.32%). Furthermore, regarding the request for consent to carry out the restraint, whether verbal or written, professionals considered obtaining consent from the family and/or guardian (n = 617; 68.78%), but 398 (44.37%) reported not requesting authorization from the child.

Regarding family involvement, nursing professionals underscored that families remained present during restraint procedures (n = 757; 84.39%), and were frequently instructed to assist in the child’s physical restraint (n = 648; 76.25%).

Most nursing professionals (n = 464; 51.73%) offered some strategy to control potential harm after the completion of a healthcare procedure involving restraint. Among the most frequently cited strategies to minimize potential harm are the comfort of a family member (n = 392; 43.70%) and positive reinforcement (n = 175; 19.51%).

Most participants stated that they record information about the restraint in the child’s medical record (n = 630; 70.23%). Information about the procedure and what required the use of restraint was most frequently recorded (n = 494; 55.07%); however, recording of the child’s consent in the medical record was less common (n = 87; 9.70%).

## DISCUSSION

The distribution of nursing professionals participating in the research showed the highest concentration in the Southeast macro-region, while the lowest number of participants was concentrated in the North and Northeast macroregions. According to data published by the Federal Nursing Council (*COFEN*)^(17)^, the state of São Paulo has the largest number of nursing professionals (664,956), followed by Rio de Janeiro (361,886) and Minas Gerais (262,394), all located in the Southeast region. The only Brazilian study that addresses the profile of nursing professionals working in the pediatric and neonatal area showed that the most frequently cited place of residence for pediatric and neonatal nurses was in the Southeast and South macro-regions^(18)^.

This study found widespread adoption of the restraint method for performing healthcare procedures on hospitalized children by Brazilian nursing professionals, especially nursing technicians. The variation in its use in the workplace among nursing team members may be related to the responsibilities of each professional category, supported since 1986 by Law No. 7,498, which regulates the practice of nursing in Brazil^(19)^. It stipulates that nursing professionals perform all nursing activities plus some functions that are exclusive to them, while nursing technicians and assistants perform, among other duties, the execution of nursing care actions, except those exclusive to nurses^(19)^. Consequently, because they are more involved in the direct care of pediatric patients and are quantitatively greater in number than nurses, these professionals may be more subject to the use of restraint.

Corroborating the findings on the frequent use of restraint by nursing staff in Brazilian pediatric care, this evidence was also found in other studies conducted internationally^(2,20,21)^. In Ireland, researchers analyzed the perceptions of 50 nurses from a children’s hospital regarding the use of restraint, and the findings revealed that 76% of them used the method on children during some clinical care^(21)^. In another setting, 30 healthcare professionals, including nurses, working in four hospitals with pediatric facilities in France, also reported that resorting to restraint in child care was a common practice^(2)^. Finally, an international investigation analyzing the perspective of healthcare professionals on child restraint practices, in which 75% were nurses, revealed that this was a very frequent practice for professionals working in Australia, New Zealand, and the United Kingdom^(20)^.

With regard to professional training and qualifications, the participating nursing team reported not having received preparatory content on restraint techniques during their training process and not having received any updates/training on the subject during their professional lives. This worrying result was also identified in research conducted in other countries, such as Ireland, where 98.5% of the nurses surveyed reported not having been trained on child restraint in the children’s hospital where they worked^(21)^.

In this context, research led by a nurse in the United Kingdom obtained results that enrich the discussion about the importance of continuing education, as it showed that 60% of the healthcare professionals participating in the investigation also reported not having received training on this topic^(20)^. For those who received it, the training was a one-off event, taking place during undergraduate or graduate studies, or in on-the-job courses^(20)^. Moreover, a statistically significant association was observed in the frequency of restraint use between trained and untrained professionals, with untrained professionals using it more frequently^(20)^.

Investing in continuing education for professionals is a strategy to ensure safe and harm-free patient care. Regarding restraint, despite being a widely adopted method by healthcare professionals in child care, little is known or discussed about its repercussions for the child subjected to this procedure^(4)^. Considering the potential for emotional distress that restraint can cause for children, healthcare professionals need to be trained to implement alternatives in their workplaces that promote positive experiences and respect children’s rights and well-being^(1,2,3)^.

Specifically regarding rights, in Brazil, the National Council for the Rights of Children and Adolescents (*CONANDA*), through Resolution No. 41/1995, established the rights of hospitalized children and adolescents^(22)^. Thus, the child is guaranteed the right to have adequate knowledge about their illness, the therapeutic care to be provided, the diagnoses and prognoses, respecting their cognitive stage, to receive psychological support when necessary, and also to the full protection of their physical, psychological, and moral dignity^(22)^.

It is worth noting that in Brazil, there are still no specific regulations or guidelines regarding the use of child restraint. The only regulation addressing the topic of restraint in the country was proposed in 2012 by *Cofen*, through resolution no. 427/2012, which specifically dealt with mechanical restraint and nursing procedures in the use of this type of restraint^(23)^. The aforementioned resolution was revoked with the publication of a new one, resolution no. 746, in March 2024^(24)^. In this update to the professional regulations, the caveat described in the second paragraph of article 4 of the previous resolution, which addressed the need for greater rigor in monitoring mechanical restraint in children and adolescents, has been removed. In this regard, Article 2 of the new resolution highlights the need for monitoring in general, to promote patient safety and prevent harm and adverse events.

To discuss the issues surrounding the use of restraint in childhood, a scoping review was undertaken to map interventions or guidelines to prevent the use of restraint during healthcare procedures in hospitalized children. This study identified 11 guidelines, policies, or recommendations with this focus, and of these, only one was specifically about restraint and aimed at healthcare professionals^(25)^. Among their recommendations, they highlighted the importance of providing training, assessment, and documentation so that professionals acquire the knowledge and develop the skills to address this issue in clinical practice^(25)^. Finally, the authors also highlighted the need to establish guidelines regarding the use of restraint in pediatrics, including definitions, the establishment of children’s rights, and the responsibilities of healthcare professionals in these situations.

With a view to ensuring children’s rights in their relationship with health services, and aiming to contribute to harm reduction during health procedures, the *iSupport collaboration* proposed the ‘Good practices based on the rights of children undergoing health procedures’, defining actions that guide health professionals in the use of restraint with support during health procedures to ensure respect for children’s rights^(1,26,27)^.

Regarding the use of restraint, in the experience of the nursing professionals participating in this study, the most frequently used technique was the physical one, and the healthcare procedure that most required its use was venipuncture. Among the reasons that justified its use, some related to the child were highlighted, such as their behavior, the need to guarantee their safety, their refusal to cooperate in carrying out the procedure; depending on the age group, the need for restraint is greater. Other investigations corroborate these findings and emphasize that, among the factors reported by health professionals as capable of influencing a child to be restrained for health procedures, the most prominent were the child’s safety (31%), age (31%), and distress (9%)^(20)^. In turn, based on the experience of nurses and doctors working in the children’s unit of a Norwegian hospital, physical and chemical restraint techniques were the most frequently used during peripheral venous puncture in preschool children^(28)^.

The literature consistently indicates that the hospitalization process can have negative impacts on a child’s emotional development^(1,4,29)^. This is because, during this period, the child is removed from their familiar environment and begins to experience unfamiliar situations often involving pain as a result of medical procedures^(1,29)^. Consequently, this experience is interpreted as stressful and generates negative feelings that can have repercussions throughout one’s life^(4,29)^.

Therefore, to minimize the feelings of this nature triggered by the child’s hospitalization, new technologies have been tested in Brazil to assist in healthcare procedures. From this perspective, virtual reality glasses were used in a public children’s hospital in southern Brazil during the insertion of peripheral intravenous catheters in pediatric emergencies and proved to be a promising strategy^(30)^. The children and family members participating in the study evaluated the use of virtual reality as a positive method for reducing negative feelings associated with undergoing catheterization. In addition, the authors highlighted that 91.6% of the children did not exhibit verbal resistance or struggle during the procedure, and 66.8% did not need to be restrained^(30)^.

Another result that stood out in this study refers to information about restraint and obtaining consent to carry it out, both from the family member and/or guardian, and from the child. In this regard, the data showed that, in practice, nursing professionals informed the family and/or guardian about restraint and obtained consent more frequently than from the child. Additionally, in Australia, New Zealand, and the United Kingdom, 76% of healthcare professionals participating in a survey reported requesting consent from the family and/or guardian before restraining a child. However, regarding the child’s own consent, most participants reported that this practice was not necessary (33%) or that they did not know (29%)^(20)^.

Considering the findings of this study on obtaining children’s consent, a brief reflection is in order. We understand that the child’s consent should be sought whenever they have the cognitive capacity to understand. In the case of a child hospitalized in the NICU, the professional must obtain consent from the responsible family member and follow the recommendations for restraint with support, which establish attitudes of respect for children’s rights^(1,26,27)^, such as: discussion among the team about the need for and the restraint technique to be adopted; establishing a bond with the child and their family; during the restraint, ensuring the child’s comfort; ensuring their well-being after the procedure is completed; and documenting information about the restraint in the medical record.

Regarding consideration for the child during care, the authors of a study conducted in France showed that, when faced with a health procedure to be performed, healthcare professionals temporarily lose this consideration^(2)^. Previously, the focus of care was the child; subsequently, it becomes the technical procedure, a process termed ‘transitory empathic blindness’^(2)^.

In this regard, two recently published scoping reviews on the phenomenon of pediatric restraint, aiming to comprehensively map the scientific knowledge, showed that the interests of children did not seem to be a priority in most of the research analyzed by the authors^(3,25)^. In both cases, the focus on children’s experiences with restraint was limited, as most studies aimed to investigate the experiences and practices of healthcare professionals and/or family members^(3,25)^. Furthermore, many studies did not include information about the child’s consent to restraint in care practice^(3)^. Therefore, it can be inferred that, in addition to children occupying less space in academic and scientific discussions about their right to be informed and to consent to procedures, this does not seem to be different in clinical practice^(25)^.

Seeing through this aspect, a study corroborates reflections on the results obtained in this study, as it analyzed the autonomy granted to children in making decisions about their healthcare in light of the normative discourse on child protection in Brazil^(31)^. The results indicated that the child’s participation in this scenario is a fundamental right, but the topic remains controversial, since the recognition of the right to dignity and freedom, information and protection depends on adults’ judgment of their capacity for discernment. Protecting children is a responsibility of the state, society, and family^(31)^.

Regarding family involvement during the child restraint process, the results of this research highlighted that the family was present during the restraint and was frequently instructed to assist in the child’s physical restraint. In this respect, the scientific literature is consistent with these findings, since in 46.7% of the articles analyzed in a scoping review on restraint, the presence of parents was reported or they were observed helping healthcare professionals restrain their children, and in 63.3% of the studies, parents were present during children’s restraint^(3)^.

In this framework, a recent study conducted in Australia, aimed at exploring the experiences of parents who helped restrain children during various hospital procedures, highlighted that they played a protective role in this context^(32)^. Thus, driven by the desire for their children to feel safe, they comforted the children and supported the healthcare professionals in carrying out the procedure. Taking into account the roles parents can assume during care, the authors recommend that parents be included during the performance of health procedures on children.

Aiming at mitigating potential harm to the child, the nursing team participating in this study highlighted that, after completing the health procedure using restraint, they used to provide some strategy to control potential harm, with holding the child in the arms and positive reinforcement being the most frequent. In clinical practice, healthcare professionals appear to be making efforts to employ, in addition to restraint, other strategies that facilitate the execution of healthcare procedures in pediatrics. From this perspective, research conducted in Norway with the aim of describing how nurses and doctors dealt with resistance from preschool children during peripheral venous puncture showed the use of different strategies^(28)^. Among these, comments were identified that aimed to acknowledge the child’s emotions and anxieties and, more commonly, to distract them through questions and compliments that diverted their attention from the technical procedure^(28)^.

Conversely, research conducted with 712 healthcare professionals working in ten different countries revealed that they sought to consider children’s choices; however, the numerous demands of the services frequently led to the use of restraint to complete the procedure more quickly^(33)^. Furthermore, participants expressed difficulty in calming and engaging the child when other strategies failed, while the procedure needed to be completed, which became a clinical dilemma to manage^(33)^.

Another result underscored in this investigation concerns the record of restraint in the child’s medical record. In this regard, data showed that professionals recorded the procedure, as well as what required the use of restraint, and the presence and authorization of a family member and/or guardian. However, the child’s consent was the least recorded piece of information in daily work. In this regard, research conducted in a children’s hospital in England highlighted, based on nonparticipant observation of 31 healthcare procedures performed on children and interviews with healthcare professionals, including nurses, that the recording of information about restraint was not routinely done by professionals in the child’s medical record^(5)^. Nevertheless, according to the best practices recommended by *iSupport collaboration*, which proposes an approach that prioritizes children’s rights and minimizes potential harm from restraint, this documentation is one of the key elements^(1,26,27)^.

It is well-known in the scientific literature that the practice of child restraint poses ethical and moral dilemmas for healthcare professionals^(1,2,3,6,28)^. Therefore, it becomes essential to develop new research aimed at understanding how healthcare professionals respond to this phenomenon in clinical care, thus making them aware of their own practices, as well as allowing them to reflect on the use of strategies that respect children’s rights, even with the challenge posed by children’s resistance to health procedures^(28)^.

Finally, it should be considered that healthcare professionals, including the nursing staff, are embedded in a formative, care-related, managerial, and health context that can impose significant restrictions on meeting the specific needs of the child and their family. Thus, it should be stressed that the practice of restraining children during healthcare procedures appears to be directly affected by the training and skills development received by professionals^(20)^. These are important aspects to be taken into account when reflecting on and justifying the necessary advancements to guarantee the rights ensured to children and, consequently, to ensure that nursing care is guided by ethical principles.

The results of this study are subject to limitations. It should be noted that the findings reported here relate to the experience of nursing professionals working in the Brazilian health system and, therefore, are limited to this healthcare setting. Furthermore, it must be considered that our country extends over continental dimensions, which can even influence nursing practices. Similarly, it should be noted that, despite the researchers’ efforts, a proportional sample was not achieved in the Northeast macro-region in the strata of nurses and nursing technicians/assistants, and in the South macro-region in the stratum of nursing technicians/assistants, which may in some way interfere with the interpretation of the findings. The convenience sampling method and recall bias should also be considered, since long-time nursing professionals may not accurately remember previous experiences.

## CONCLUSION

The restraint method has become a common practice for nursing staff to perform procedures such as venipuncture, medication administration, and catheterization in hospitalized children, with physical restraint, supported restraint, and psychological restraint being the most frequently used techniques. Its use was justified by the child’s behavior, the need to ensure their safety, their refusal to cooperate, and also by their age. Likewise, nursing professionals prioritize the family and/or guardian over the child when explaining the restraint procedure and obtaining consent to perform it. After the restraint was performed, it was common to offer a hug from a family member and positive reinforcement to mitigate any potential harm. In the child’s medical record, professionals documented information about the health procedure and what required the use of restraint, as well as the presence and consent of the family and/or guardian. However, the professionals’ accounts revealed a lack of preparedness among the nursing team due to the absence of content on the subject in their training and professional development/updating throughout their careers.

In light of the results, it is noteworthy that these could serve as a basis for the development of national guidelines, professional standardization by the nursing council, and the development of institutional protocols. These documents should stipulate recommendations for the nursing staff regarding the aspects involved in the practice of restraining hospitalized children and the ethical dilemmas faced in clinical care. Furthermore, they may promote the incorporation of this content and its specificities into the teaching and learning process during nursing education in the country, both at the technical level and in undergraduate and graduate studies.

Ultimately, it is crucial to highlight the need to broaden the scope of this research topic in Brazil from different perspectives. Therefore, further scientific research is encouraged that considers restraint in various care contexts, in addition to ensuring the child’s protagonism. This will allow the phenomenon to be mapped comprehensively, diversely, and in its various peculiarities, with the purpose of advancing nursing practices with respect and consideration for the children’s emotions and rights.

## Data Availability

All the data supporting the results of this study were published in the article itself.
