# Supplementary material for: Restraining hospitalized children for procedures: practices of nursing professionals in Brazil
Source: Rev Esc Enferm USP. 2026 May 29;60:e20250453. doi: 10.1590/1980-220X-REEUSP-2025-0453en (PMC13225314; doi:10.1590/1980-220X-REEUSP-2025-0453en)
Supplement: Translation

# Translation: Contenção da criança hospitalizada para a realização de procedimentos: práticas dos profissionais de enfermagem no Brasil*

**DOI:** 10.1590/1980-220X-REEUSP-2025-0453pt

**Authors:** Ronaldo Antonio da Silva, Maria Aparecida Munhoz Gaíva

**Affiliations:** 1 Universidade do Estado de Mato Grosso, Diamantino, MT, Brasil.; 2 Universidade do Estado de Mato Grosso, Diamantino, MT, Brasil.

**Keywords:** Criança Hospitalizada, Procedimentos Clínicos, Restrição Física, Equipe de Enfermagem, Cuidados de Enfermagem

## Abstract

**Objetivo:**

Descrever as práticas de contenção empregadas por profissionais de enfermagem brasileiros ao realizar procedimentos de saúde na criança hospitalizada.

**Método:**

Estudo transversal conduzido com 897 profissionais que atuavam no cuidado à criança hospitalizada no país. Os dados foram coletados entre maio/2024 e janeiro/2025, utilizando-se um questionário com informações sociodemográficas e outro sobre a prática de contenção, submetido à validação de conteúdo e pré-teste. O banco foi analisado no *software R.*

**Resultados:**

A contenção caracterizou-se como um método usual; todavia, os profissionais relataram que não receberam preparação durante a formação e/ou atuação profissional para executá-la. As técnicas mais utilizadas foram a física, com apoio e psicológica, nos procedimentos de punção venosa, administração de medicamentos e sondagens. A opção de empregá-la relacionava-se ao comportamento da criança, à garantia de sua segurança e à sua recusa em cooperar.

**Conclusão:**

As práticas de contenção utilizadas pela enfermagem brasileira para o cuidado pediátrico foram descritas com base em técnicas, motivos, consentimento, participação familiar, controle de possíveis danos e registro em prontuário.

## INTRODUÇÃO

A contenção é definida como qualquer método que limite o movimento, a atividade física ou o acesso normal de uma pessoa ao seu corpo, e tem sido frequentemente adotada por profissionais de saúde na assistência pediátrica^(1,2,3)^. Tal prática é utilizada durante a hospitalização da criança para diferentes procedimentos de saúde, tais como: punção venosa, administração de medicamentos, sondagens, exames de sangue e radiológicos^(1,2,3)^. Todavia, a contenção da criança é envolta por incertezas e controvérsias a respeito das implicações éticas e morais relacionadas à atuação profissional, além dos riscos e consequências de curto, médio e longo prazo na vida e saúde das crianças que são contidas^(3,4)^.

Sabe-se que a prática da contenção durante a realização de procedimentos de saúde pode ter efeitos prejudiciais para a criança, conforme apontam as evidências científicas. Pesquisadores suecos, ao analisarem as narrativas das crianças que passavam por diferentes procedimentos de saúde não urgentes, destacaram que, para essas crianças, a contenção significava sofrimento emocional^(4)^. Nesta pesquisa, as crianças relataram que se sentiam magoadas ao serem obrigadas a passar por tais procedimentos, e que as lembranças desses momentos permaneciam por muito tempo. Além disso, de acordo com a inferência dos autores, essas memórias negativas podem afetar a interação da criança com os profissionais em futuros serviços de saúde.

Neste sentido, sabe-se que a angústia antecipada é uma realidade na vida de crianças hospitalizadas e submetidas a procedimentos de saúde. De acordo com a literatura, elas podem experimentar angústia tanto antes dos procedimentos quanto ao vivenciarem a contenção contra a sua vontade^(1,4,5,6)^. Dentre os fatores que desencadeiam e potencializam o sentimento de angústia antecipada antes de um procedimento de saúde está a experiência negativa das crianças e dos familiares em procedimentos prévios e hospitalizações^(7)^.

Quanto à aplicação da contenção na prática, uma recente revisão de escopo destacou que, dentre os profissionais de saúde, os que compõem a equipe de enfermagem foram citados em 61,5% dos estudos como aqueles envolvidos na contenção das crianças^(3)^. Além disso, foram catalogados estudos realizados em diferentes países, com destaque para os do continente europeu. Entretanto, não foram identificados estudos publicados sobre esse assunto no Brasil, o que indicou uma lacuna na literatura científica no país^(3)^. Diante da problemática apresentada, levantou-se a seguinte pergunta de pesquisa: quais são as práticas de contenção utilizadas pelos profissionais de enfermagem brasileiros para a execução de procedimentos de saúde na criança hospitalizada?

Esta pesquisa tem o potencial de se aproximar das práticas diárias dos profissionais de enfermagem nos serviços de saúde do Brasil e, assim, compreender como o fenômeno da contenção de crianças hospitalizadas se apresenta neste contexto social e cultural, para o qual ainda há poucos dados disponíveis. Esses resultados contribuirão para a literatura científica internacional, na perspectiva de ampliar o escopo para subsidiar abordagens que priorizem a garantia dos direitos e assegurem o bem-estar físico, emocional e psicológico das crianças durante a realização de procedimentos de saúde não urgentes. Assim, este estudo teve como objetivo descrever as práticas de contenção empregadas por profissionais de enfermagem brasileiros ao realizar procedimentos de saúde na criança hospitalizada.

## MÉTODO

### Tipo de Estudo

Trata-se de um estudo transversal.

### Local, População, Critérios de Seleção e Amostra

O local do estudo foi o Brasil, país dividido em cinco macrorregiões para fins de organização político-administrativa, Norte, Nordeste, Sul, Sudeste e Centro-Oeste, que englobam as 27 Unidades da Federação^(8)^.

A população foi composta por enfermeiros(as), técnicos(as) e/ou auxiliares de enfermagem que atuavam no cuidado à criança hospitalizada nas cinco macrorregiões do país. A amostragem ocorreu por autosseleção, uma técnica não probabilística empregada quando os participantes têm características relevantes para a pesquisa e se oferecem como voluntários para participar^(9)^. Neste estudo, o critério de inclusão era possuir experiência profissional de no mínimo seis meses na assistência de enfermagem pediátrica e/ou neonatológica, tempo este considerado adequado para garantir a familiaridade e vivência com as práticas de contenção da criança hospitalizada, e foram excluídos os profissionais que participaram do pré-teste.

Em relação ao tamanho da amostra, ao utilizar os dados da pesquisa sobre o perfil da enfermagem no Brasil^(10)^, realizou-se o cálculo de amostragem proporcional para cada macrorregião brasileira nos estratos de enfermeiros(as) e técnicos(as) e auxiliares de enfermagem. Com um nível de confiança de 95%, era necessária uma amostra mínima de 385 sujeitos para cada estrato. A amostra alcançada foi composta por 897 profissionais de enfermagem.

### Coleta de Dados

A coleta dos dados ocorreu entre maio de 2024 e janeiro de 2025. Foram utilizados: um questionário com questões referentes às informações sociodemográficas e outro sobre a experiência com a contenção nos procedimentos de saúde na criança durante a hospitalização. Este segundo foi elaborado com base em uma revisão de escopo sobre o tema^(3)^ e submetido a validação de conteúdo para alcançar legitimidade e credibilidade. Participaram dessa etapa seis especialistas, sendo três docentes e pesquisadoras, uma enfermeira supervisora da pediatria, uma enfermeira assistencial da pediatria e uma enfermeira assistencial na Unidade de Terapia Intensiva Neonatal (UTIN). Após a revisão dos domínios e itens, este questionário apresentou o índice de Validade de Conteúdo (IVC) de 0,86, considerado um nível de concordância satisfatório^(11,12)^.

Assim, foram investigadas variáveis atinentes às características sociodemográficas (categoria profissional, idade, sexo, estado civil, presença de filho e macrorregião de residência); formação profissional (ter estudado ou feito curso recente sobre contenção, possuir ou cursar pós-graduação na área pediátrica/neonatológica e a modalidade); atuação (área e tempo de trabalho); e práticas de contenção (uso, quem realiza, técnica, procedimento associado, motivos, percepção de necessidade por idade/sexo, comunicação/consentimento com familiar e criança, presença/ajuda do familiar, controle de danos e registro em prontuário, incluindo o tipo de informação registrada).

Depois desse processo, os questionários foram cadastrados no *Google Forms* ^®^ e submetidos ao pré-teste, sendo aplicados a nove profissionais de enfermagem que trabalhavam em unidade de pronto atendimento com a oferta de assistência de urgência e emergência a recém-nascidos, neonatos e/ou crianças no estado de Mato Grosso, macrorregião Centro-Oeste. Posteriormente, o *link* com os questionários e o Termo de Consentimento Livre e Esclarecido (TCLE) foram enviados aos participantes por *e-mail*, redes sociais (*Instagram* ^®^ e/ou *Facebook* ^®^), *WhatsApp* ^®^. Além disso, o *link* foi compartilhado nas cinco macrorregiões do país com apoio das entidades de classe da profissão, instituições educacionais, grupos de pesquisas e hospitais com atendimento pediátrico.

### Análise e Tratamento dos Dados

O banco de dados foi gerado em uma planilha no *Microsoft Excel* ^®^, que passou por revisão dos pesquisadores para assegurar consistência e integridade. Adicionalmente, as colunas foram renomeadas para facilitar o processamento no software de análise. Após isso, o banco foi analisado no software R^(13)^, com cálculo das frequências absolutas e relativas para as variáveis qualitativas e para as quantitativas, as medidas de tendência central e dispersão, incluindo média, desvio padrão, valores mínimos e máximos.

### Aspectos Éticos

Esta pesquisa respeitou os preceitos éticos de pesquisas com seres humanos no Brasil e foi aprovada pelo Comitê de Ética em Pesquisa da Universidade Federal de Mato Grosso (CEP-SAÚDE/UFMT) sob o parecer n^o^ 6.672.454/2024 e CAAE n^o^ 76648023.4.0000.8124, de acordo com as resoluções n^o^ 466/2012 e 510/2016 do Conselho Nacional de Saúde^(14,15)^. Ademais, seguiu as diretrizes estabelecidas no ofício circular n^o^ 2/2021 da Comissão Nacional de Ética em Pesquisa (CONEP), que orienta os procedimentos em pesquisas com qualquer etapa em ambiente virtual no país^(16)^.

## RESULTADOS

Participaram deste estudo 897 profissionais de enfermagem, cujas características sociodemográficas, de formação e atuação estão apresentadas na Tabela 1. A maior proporção dos profissionais residia na macrorregião Sudeste (n = 558; 62,21%), seguida pela Centro-Oeste (n = 113; 12,60%), Sul (n = 91; 10,14%), Nordeste (n = 68; 7,58%) e Norte (n = 67; 7,47%). Em relação à categoria profissional, a maior parte era de enfermeiros(as) (n = 462; 51,51%), seguida por técnicos(as) de enfermagem (n = 349; 38,91%) e auxiliares de enfermagem (n = 86; 9,59%). A idade dos participantes variou entre 18 e 72 anos, com média de 40,9 anos e desvio padrão de 10,12 anos, indicando uma faixa etária diversificada. A grande maioria dos respondentes era do sexo feminino (n = 785; 87,51%) e, em termos de estado civil, a maior parte declarou-se casado(a) (n = 472; 52,62%) e, no que diz respeito à presença de filhos, 627 profissionais (69,90%) afirmaram ter filhos.

**Tabela 1:** Características sociodemográficas, de formação e atuação dos profissionais de enfermagem estudados – Cuiabá, MT, Brasil, 2025.

Variáveis	n (%)	Média (DP)
Categoria profissional		
Enfermeiro(a)	462 (51,51%)	
Técnico(a) de enfermagem	349 (38,91%)	
Auxiliar de enfermagem	86 (9,59%)	
Idade (anos)		40,87 (10,12)
Sexo		
Masculino	112 (12,49%)	
Feminino	785 (87,51%)	
Estado civil		
Solteiro(a)	326 (36,34%)	
Casado(a)	472 (52,62%)	
Divorciado(a)	90 (10,03%)	
Viúvo(a)	9 (1,0%)	
Tinha filho(s)		
Sim	627 (69,90%)	
Não	270 (30,10%)	
Macrorregião de residência		
Norte	67 (7,47%)	
Nordeste	68 (7,58%)	
Centro-Oeste	113 (12,60%)	
Sudeste	558 (62,21%)	
Sul	91 (10,14%)	
Estudou conteúdo sobre contenção durante a formação		
Sim	285 (31,77%)	
Não	612 (68,23%)	
Fez curso de capacitação ou atualização sobre contenção nos últimos 12 meses		
Sim	81 (9,03%)	
Não	816 (90,97%)	
Possuía pós-graduação na área pediátrica/neonatológica		
Sim	227 (25,31%)	
Não	670 (74,69%)	
Modalidade da pós-graduação na área		
Residência	22 (9,69%)	
Especialização	150 (66,08%)	
Mestrado profissional	9 (3,96%)	
Mestrado acadêmico	19 (8,37%)	
Doutorado	20 (8,81%)	
Pós-doutorado	7 (3,08%)	
Estava cursando pós-graduação	na área	
Sim	67 (7,47%)	
Não	830 (92,53%)	
Modalidade da pós-graduação em curso	
Residência	10 (14,93%)	
Especialização	35 (52,24%)	
Mestrado profissional	1 (1,49%)	
Mestrado acadêmico	6 (8,96%)	
Doutorado	14 (20,90%)	
Pós-doutorado	1 (1,49%)	
Área de atuação profissional^*^		
Saúde do recém-nascido	408 (45,48%)	
Saúde da criança	588 (65,55%)	
Saúde do adolescente	491 (54,74%)	
Tempo de trabalho (anos)		7,2 (8,69)

*O participante poderia assinalar mais de uma opção; DP: Desvio Padrão.

Quanto à formação, 612 (68,23%) profissionais relataram não lhes ter sido oferecido nenhum conteúdo relativo à contenção da criança durante a graduação ou o curso técnico/auxiliar de enfermagem. No que se refere à educação continuada, 816 (90,97%) afirmaram não ter participado de curso de capacitação ou atualização sobre contenção de crianças nos últimos 12 meses. Ainda sobre a formação, 227 profissionais (25,31%) indicaram possuir algum tipo de pós-graduação na área pediátrica ou neonatológica, sendo que a especialização foi a modalidade mais recorrente (n = 150; 66,08%), seguida pela residência (n = 22; 9,69%), pelo doutorado (n = 20; 8,81%), mestrado acadêmico (n = 19; 8,37%), mestrado profissional (n = 9; 3,96%) e pós-doutorado (n = 7; 3,08%). No momento da coleta, 67 profissionais (7,47%) estavam cursando alguma pós-graduação nestas áreas; a especialização representou novamente a principal modalidade (n = 35; 52,24%), seguida do doutorado (n = 14; 20,9%) e residência (n = 10; 14,93%).

No que se refere à área de atuação profissional, os participantes puderam assinalar múltiplas áreas e a maioria atuava na saúde da criança (n = 588; 65,55%), seguida por aqueles que atuavam com a saúde do adolescente (n = 491; 54,74%) e com a saúde do recém-nascido (n = 408; 45,48%). Quanto ao tempo de trabalho na enfermagem pediátrica e/ou neonatológica, os dados apontaram uma variação entre 1 e 50 anos de experiência (7,2 ± 8,69).

A Tabela 2 destaca as práticas de contenção empregadas pelos profissionais de enfermagem brasileiros em procedimentos de saúde na criança hospitalizada. A maioria dos profissionais de enfermagem (n = 760; 80,73%) relatou já ter utilizado o método de contenção para efetuar procedimentos de saúde na criança hospitalizada, evidenciando uma ampla adoção desta prática no contexto hospitalar. Foi constatado, ainda, que os técnicos(as) de enfermagem (n = 647; 72,13%) foram referidos como os que mais realizavam a contenção no ambiente de trabalho.

**Tabela 2:** Práticas de contenção utilizadas pelos profissionais de enfermagem brasileiros para realizar procedimentos de saúde na criança hospitalizada – Cuiabá, MT, Brasil, 2025.

Variáveis	n (%)
Utilizou a contenção	
Sim	760 (84,73%)
Não	137 (15,27%)
Profissional da enfermagem que mais realiza a contenção no seu local de trabalho	
Enfermeiro(a)	175 (19,51%)
Técnico(a) de enfermagem	647 (72,13%)
Auxiliar de enfermagem	75 (8,36%)
Técnica utilizada em seu serviço^*^	
Física	
Sim	590 (65,77%)
Não	307 (34,23%)
Mecânica	
Sim	257 (28,65%)
Não	640 (71,35%)
Química	
Sim	202 (22,52%)
Não	695 (77,48%)
Psicológica	
Sim	349 (38,91%)
Não	548 (61,09%)
Contenção com apoio	
Sim	441 (49,16%)
Não	456 (50,84%)
Procedimento de saúde^*^	
Administração de medicamentos	
Sim	448 (49,94%)
Não	449 (50,06%)
Curativo	
Sim	272 (30,32%)
Não	625 (69,68%)
Imunização	
Sim	255 (28,43%)
Não	642 (71,57%)
Punção venosa	
Sim	795 (88,63%)
Não	102 (11,37%)
Sondagens	
Não	384 (42,81%)
Sim	513 (57,19%)
Motivo(s) para utilizar a contenção^*^	
Idade	
Sim	343 (38,24%)
Não	554 (61,76%)
Comportamento (agitada/agressiva)	
Sim	731 (81,49%)
Não	166 (18,51%)
Recusa em cooperar	
Sim	476 (53,08%)
Não	421 (46,93%)
Presença de alguma deficiência	
Sim	228 (25,42%)
Não	669 (74,58%)
Capacidade de compreensão	
Sim	429 (47,83%)
Não	468 (52,17%)
Garantir a segurança da criança	
Sim	720 (80,27%)
Não	177 (19,73%)
Necessidade de concluir o procedimento	
Sim	460 (51,28%)
Não	437 (48,72%)
Tempo limitado para realizar o procedimento	
Sim	192 (21,40%)
Não	705 (78,60%)
Contenção pela faixa etária	
Sim	803 (89,52%)
Não	94 (10,48%)
Contenção pelo sexo	
Sim	268 (29,88%)
Não	629 (70,12%)
Informava o familiar e/ou responsável	
Sim	814 (90,75%)
Não	31 (3,46%)
Às vezes	52 (5,8%)
Informava à criança	
Sim	568 (63,32%)
Não	105 (11,71%)
Às vezes	224 (24,97%)
Solicitava consentimento do familiar e/ou responsável	
Sim	617 (68,78%)
Não	191 (21,29%)
Às vezes	89 (9,92%)
Solicitava consentimento da criança	
Sim	304 (33,89%)
Não	398 (44,37%)
Às vezes	195 (21,74%)
O familiar fica presente no momento da contenção	
Sim	757 (84,39%)
Não	24 (2,68%)
Às vezes	116 (12,93%)
O familiar é orientado a ajudar a realizar a contenção física da criança	
Sim	648 (76,25%)
Não	64 (7,13%)
Às vezes	149 (16,61%)
	continuar...
Controle dos possíveis danos	
Sim	464 (51,73%)
Não	227 (25,31%)
Às vezes	206 (22,97%)
Estrategias de controle^*^	
Colo do familiar	
Sim	392 (43,70%)
Não	505 (56,30%)
Pomada para evitar hematomas	
Sim	51 (5,69%)
Não	846 (94,31%)
Reforço positivo (certificados e presente)	
Sim	175 (19,51%)
Não	722 (80,49%)
Outros	
Sim	56 (6,24%)
Não	841 (93,76%)
Registrava no prontuário	
Sim	630 (70,23%)
Não	161 (17,95%)
Às vezes	106 (11,82%)
Informações registradas^*^	
Procedimento e o que demandou o uso da contenção	
Sim	494 (55,07%)
Não	403 (44,93%)
Tipo de contenção utilizada	
Sim	248 (27,65%)
Não	649 (72,35%)
Profissional de saúde que ajudou a conter a criança	
Sim	153 (17,06%)
Não	744 (82,94%)
Presença do familiar/responsável	
Sim	262 (29,21%)
Não	635 (70,79%)
Consentimento do familiar/responsável	
Sim	262 (29,21%)
Não	635 (70,79%)
Consentimento da criança	
Sim	87 (9,70%)
Não	810 (90,30%)
Aspectos que colaboraram com a decisão de conter	
Sim	185 (20,62%)
Não	712 (79,38%)

*O participante poderia assinalar mais de uma opção.

Em relação à técnica de contenção utilizada pelos profissionais, a contenção física foi a mais frequente (n = 590; 65,77%), seguida pela contenção com apoio (n = 441; 49,16%) e a contenção psicológica (n = 349; 38,91%). Quanto aos procedimentos de saúde em que a contenção era mais frequentemente utilizada pelos profissionais de enfermagem, destacaram-se: a punção venosa (n = 795; 88,63%), a administração de medicamentos (n = 448; 49,94%) e sondagens (n = 384; 42,81%).

Os motivos mencionados pelos profissionais de enfermagem para o uso da contenção na criança hospitalizada foram: o comportamento da criança (n = 731; 81,49%), garantir a sua segurança (n = 720; 80,27%) e, ainda, a sua recusa em cooperar (n = 476; 53,08%). Quanto à necessidade de contenção, a maioria dos profissionais (n = 629; 70,12%) não identificou diferença em razão do sexo das crianças.

Ao analisar os dados sobre a explicação do tipo de contenção adotada, observou-se que a maior proporção dos profissionais de enfermagem considerava informar a família e/ou o responsável (n = 814; 90,75%) em detrimento da criança (n = 568; 63,32%). Além disso, no que tange à solicitação de consentimento para efetuar a contenção, seja verbal ou escrita, os profissionais consideravam obter o consentimento da família e/ou o responsável (n = 617; 68,78%), mas 398 (44,37%) relataram não solicitar autorização para a criança.

Ainda sobre a participação da família, os profissionais de enfermagem ressaltaram que ela permanecia presente no momento da contenção (n = 757; 84,39%), sendo frequentemente orientada a auxiliar na contenção física da criança (n = 648; 76,25%).

A maioria dos profissionais de enfermagem (n = 464; 51,73%) oferecia alguma estratégia para controlar possíveis danos depois de concluído o procedimento de saúde em que havia contenção. Dentre as estratégias mais citadas para minimizar os possíveis danos estão o colo do familiar (n = 392; 43,70%) e o reforço positivo (n = 175; 19,51%).

A maioria dos participantes afirmou fazer o registro das informações sobre a contenção no prontuário da criança (n = 630; 70,23%). E as informações sobre o procedimento e o que exigia o uso da contenção foram os mais frequentemente registrados (n = 494; 55,07); entretanto, o registro no prontuário de obtenção do consentimento da criança era menos comum (n = 87; 9,70%).

## DISCUSSÃO

A distribuição dos profissionais de enfermagem participantes da pesquisa teve a maior concentração na macrorregião Sudeste, enquanto o menor número de participantes estava concentrado nas macrorregiões Norte e Nordeste. Conforme dados publicados pelo Conselho Federal de Enfermagem (COFEN)^(17)^, o estado de São Paulo possui o maior quantitativo de profissionais de enfermagem (664.956), seguido pelo Rio de Janeiro (361.886) e Minas Gerais (262.394), todos localizados no Sudeste. O único estudo brasileiro que trata do perfil dos profissionais de enfermagem que atuam na área pediátrica e neonatal mostrou que o local de residência dos enfermeiros pediatras e neonatologistas mais citado estava nas macrorregiões Sudeste e Sul^(18)^.

Neste estudo, verificou-se ampla adoção do método de contenção para a realização de procedimentos de saúde em crianças hospitalizadas pelos profissionais de enfermagem brasileiros, sobretudo pelos técnicos(as) de enfermagem. A variação da sua utilização no ambiente de trabalho entre os membros da equipe de enfermagem pode estar relacionada com as atribuições de cada categoria profissional, respaldadas desde 1986 pela Lei nº 7.498, que regulamenta o exercício da enfermagem no Brasil^(19)^. Nela, determina-se que os profissionais enfermeiros exerçam todas as atividades de enfermagem somadas com algumas funções que lhes são privativas, enquanto o técnico e auxiliar de enfermagem exerçam, dentre outras atribuições, a execução das ações assistenciais da enfermagem, exceto as privativas do enfermeiro^(19)^. Consequentemente, por estarem mais presentes nos cuidados diretos aos pacientes pediátricos e quantitativamente em número maior que os enfermeiros, esses profissionais podem estar mais sujeitos à utilização da contenção.

Corroborando os achados sobre o uso frequente da contenção pela enfermagem na assistência pediátrica brasileira, também houve essa evidência em outros estudos conduzidos em âmbito internacional^(2,20,21)^. Na Irlanda, pesquisadores analisaram a percepção de 50 enfermeiras de um hospital infantil sobre o uso da contenção, e os achados revelaram que 76% delas utilizaram o método nas crianças durante algum atendimento clínico^(21)^. Em outro contexto, 30 profissionais de saúde, dentre eles enfermeiras, que atuavam em quatro hospitais com instalações pediátricas na França, também relataram que recorrer à contenção para o cuidado da criança era uma prática comum^(2)^. E, por fim, em uma investigação internacional que analisou a perspectiva de profissionais de saúde sobre as práticas de contenção da criança, em que 75% eram enfermeiros, evidenciou-se que se tratava de uma prática muito frequente para os profissionais que atuavam na Austrália, Nova Zelândia e Reino Unido^(20)^.

No que diz respeito à formação e qualificação profissional, a equipe de enfermagem participante informou não ter recebido conteúdos preparatórios sobre a contenção no processo formativo e, além disso, não receberam atualização/capacitação sobre o tema durante a vida profissional. Esse resultado preocupante também foi identificado em pesquisas realizadas em outros países, como na Irlanda, em que 98,5% das enfermeiras investigadas relataram não terem sido treinadas sobre a contenção da criança no hospital infantil em que atuavam^(21)^.

Nesse contexto, pesquisa liderada por uma enfermeira do Reino Unido obteve resultados que enriquecem a discussão sobre a importância da educação permanente, já que mostrou que 60% dos profissionais de saúde participantes da investigação também relataram não ter recebido treinamento sobre essa temática^(20)^. Para os que receberam, o treinamento foi pontual, ocorrendo na graduação, pós-graduação ou em cursos no ambiente de trabalho^(20)^. Além disso, observou-se uma associação estatisticamente significativa na frequência do uso da contenção entre os profissionais treinados e os não treinados, sendo que os não treinados a utilizavam com mais frequência^(20)^.

O investimento em educação permanente do profissional é uma estratégia para garantir um cuidado seguro e livre de danos ao paciente. No que concerne à contenção, apesar de ser um método largamente adotado pelos profissionais de saúde nos cuidados à criança, pouco se sabe ou se discute sobre suas repercussões para a criança submetida a esse procedimento^(4)^. Considerando o potencial que a contenção tem de acarretar sofrimento emocional para a criança, os profissionais de saúde precisam ser capacitados para implementar, em seus locais de trabalho, alternativas que promovam experiências positivas e respeitem os direitos e o bem-estar das crianças^(1,2,3)^.

Especificamente a respeito dos direitos, no Brasil, o Conselho Nacional dos Direitos da Criança e do Adolescente (CONANDA), a partir da Resolução nº 41/1995, estabeleceu os direitos das crianças e dos adolescentes hospitalizados^(22)^. Assim, é assegurado à criança o direito de ter conhecimento adequado sobre a sua enfermidade, os cuidados terapêuticos a serem prestados, os diagnósticos e prognósticos, respeitando sua fase cognitiva, de receber amparo psicológico, quando se fizer necessário e, ainda, à proteção integral de sua dignidade física, psíquica e moral^(22)^.

Cabe ressaltar que ainda não dispomos na realidade brasileira de nenhuma normatização ou diretriz específica sobre o uso da contenção na criança. A única normativa que trata sobre o tema da contenção no país foi proposta em 2012 pelo COFEN, por meio da resolução n^o^ 427/2012, que tratava especificamente sobre a contenção mecânica e os procedimentos da enfermagem no emprego desse tipo de contenção^(23)^. A referida resolução foi revogada com a publicação de uma nova, a resolução n^o^ 746, em março de 2024^(24)^. Nesta atualização da normatização profissional, a ressalva, descrita no inciso segundo do artigo 4^o^ da antiga resolução, que tratava sobre a necessidade de maior rigor no monitoramento da contenção mecânica em crianças e adolescentes, foi retirada. A esse respeito, o artigo 2^o^ da nova resolução destaca a necessidade de monitoramento de maneira geral, para promover a segurança do paciente e prevenir danos e eventos adversos.

Para debater as questões da utilização da contenção na infância foi empreendida uma revisão de escopo visando mapear intervenções ou diretrizes para prevenir o uso da contenção durante os procedimentos de saúde nas crianças hospitalizadas. Nesse estudo, foram constatadas 11 diretrizes, políticas ou recomendações que tinham esse enfoque, e, destas, apenas uma era específica sobre a contenção e destinada aos profissionais de saúde^(25)^. Dentre as suas recomendações, havia destaque para a importância da oferta de treinamentos, avaliação e documentação, para que os profissionais adquiram conhecimento e desenvolvam habilidades para abordar essa questão na prática clínica^(25)^. E, por fim, os autores salientaram ainda a necessidade do estabelecimento de diretrizes relativas à utilização da contenção na pediatria, com a inclusão de definições, o estabelecimento dos direitos das crianças, bem como as responsabilidades dos profissionais de saúde nessas situações.

Na perspectiva de assegurar os direitos das crianças na relação com os serviços de saúde, visando contribuir com a redução de danos durante a realização de procedimentos de saúde, o *iSupport collaboration* propôs as ‘Boas práticas baseadas em direitos das crianças submetidas a procedimentos de saúde’, definindo ações que norteiam os profissionais de saúde para o uso da contenção com apoio durante a realização de procedimentos de saúde para garantir, assim, o respeito aos direitos das crianças^(1, 26, 27)^.

Em se tratando da utilização da contenção, na experiência dos profissionais de enfermagem participantes deste estudo, a técnica de contenção mais empregada foi a física e o procedimento de saúde que mais exigiu a sua utilização foi a punção venosa. Dentre os motivos que justificaram a sua utilização, foram evidenciados alguns relativos à criança, tal como o seu comportamento, necessidade de garantir a sua segurança, a sua recusa em cooperar para a realização do procedimento e, ainda, a depender da faixa etária, a necessidade de contenção é maior. Outras investigações corroboram esses achados e salientam que, dentre os fatores relatados pelos profissionais de saúde como capazes de influenciar uma criança a ser contida para a realização de procedimentos de saúde, destacaram-se a segurança da criança (31%), a idade (31%) e a angústia (9%)^(20)^. Por sua vez, na experiência de enfermeiros e médicos que atuavam na unidade infantil de um hospital norueguês, as técnicas de contenção física e química foram as mais empregadas durante a punção venosa periférica de crianças em idade pré-escolar^(28)^.

A literatura aponta consistentemente que o processo de hospitalização pode acarretar impactos negativos para o desenvolvimento emocional da criança^(1,4,29)^. Isso porque, durante esse período, ela é retirada de seu ambiente familiar e passa a vivenciar situações desconhecidas que frequentemente envolvem dor em decorrência dos procedimentos de saúde^(1,29)^. Consequentemente, essa experiência é interpretada como estressante e gera sentimentos negativos que podem repercutir ao longo de toda a sua vida^(4,29)^.

Assim, no sentido de minimizar os sentimentos dessa ordem desencadeados pela hospitalização da criança, novas tecnologias têm sido testadas no Brasil para auxiliar nos procedimentos de saúde. Nessa perspectiva, foram utilizados em um hospital público infantil do sul do Brasil os óculos de realidade virtual durante a inserção do cateterismo intravenoso periférico em emergências pediátricas e mostraram ser uma estratégia promissora^(30)^. As crianças e os familiares participantes da pesquisa avaliaram o uso da realidade virtual como um método positivo para a redução dos sentimentos negativos vinculados à realização do cateterismo. Além disso, os autores sublinharam que 91,6% das crianças não apresentaram resistência verbal e nem se debateram durante o procedimento e 66,8% não precisaram ser contidas^(30)^.

Outro resultado que se destacou no presente estudo refere-se à informação sobre a contenção e a obtenção do consentimento para realizá-la, tanto do familiar e/ou o responsável, como da criança. Acerca disso, os dados mostraram que, na prática, os profissionais de enfermagem, informavam sobre a contenção e obtinham o consentimento com maior frequência da família e/ou responsável do que da criança. Adicionalmente, na Austrália, Nova Zelândia e Reino Unido, 76% dos profissionais de saúde participantes de uma pesquisa relataram solicitar o consentimento da família e/ou responsável antes de a criança ser contida. Entretanto, quanto ao consentimento da própria criança, a maioria dos participantes informou que esta prática não era necessária (33%) ou então que não sabia dizer (29%)^(20)^.

Considerando os achados desse estudo sobre a obtenção do consentimento da criança, cabe aqui uma breve reflexão. Entendemos que o consentimento da criança deve ser solicitado sempre que ela tiver capacidade cognitiva para compreender. No caso da criança hospitalizada na UTIN, o profissional deve solicitar o consentimento para o familiar responsável e seguir as recomendações da contenção com apoio, que estabelecem atitudes de respeito aos direitos das crianças^(1,26,27)^, tais como: discussão entre a equipe sobre a necessidade e a técnica de contenção a ser adotada; estabelecer vínculo com a criança e sua família; durante a contenção, cuidar do conforto da criança; assegurar-lhe o bem estar depois da conclusão do procedimento; e documentar no prontuário informações sobre a contenção.

Sobre a consideração pela criança durante a assistência, os autores de uma pesquisa conduzida na França mostraram que, ao se deparar com um procedimento de saúde a ser realizado, o profissional de saúde perde temporariamente essa consideração^(2)^. Antes, o sujeito do cuidado era a criança, na sequência passa a ser o procedimento técnico, denominando esse processo de ‘cegueira empática transitória’^(2)^.

Quanto a isso, recentemente, duas revisões de escopo publicadas sobre o fenômeno da contenção pediátrica, visando mapear de forma abrangente o conhecimento científico, evidenciaram que os interesses das crianças pareciam não ter prioridade na maioria das pesquisas analisadas pelos autores^(3, 25)^. Em ambos os casos, o enfoque para as vivências das crianças com a contenção era limitado, pois a maior parte dos estudos objetivava investigar as experiências e práticas dos profissionais de saúde e/ou da família^(3, 25)^. Além disso, muitas pesquisas não incluíam informações sobre o consentimento da criança para a contenção na prática assistencial^(3)^. Logo, pode-se inferir que, além de as crianças ocuparem menos espaço nas discussões acadêmicas e científicas sobre o direito de estarem informadas e consentirem com as condutas, na prática clínica isso não parece ser diferente^(25)^.

Ainda sobre este aspecto, uma investigação corrobora reflexões para os resultados obtidos na presente pesquisa, uma vez que analisou a autonomia garantida à criança na tomada de decisão sobre os seus cuidados em saúde à luz do discurso normativo de proteção à infância no Brasil^(31)^. Os resultados assinalaram que a participação da criança neste cenário é um direito fundamental, mas o tema ainda é controverso, uma vez que o reconhecimento do direito à dignidade e liberdade, informação e proteção depende do julgamento dos adultos sobre a sua capacidade de discernimento. Proteger a criança é uma responsabilidade do Estado, da sociedade e família^(31)^.

Quanto à participação da família durante a execução da contenção da criança, os resultados desta pesquisa destacaram que ela ficava presente no momento da contenção e, ainda, frequentemente era orientada a ajudar na contenção física da criança. A esse respeito, a literatura científica está em consonância com esses achados, uma vez que em 46,7% dos artigos analisados em uma revisão de escopo sobre a contenção, a presença dos pais foi relatada ou estes foram observados ajudando os profissionais de saúde a conter os seus filhos, bem como em 63,3% das produções estes estavam presentes durante a contenção das crianças^(3)^.

Nesse sentido, uma pesquisa realizada recentemente na Austrália com o objetivo de explorar as experiências dos pais que ajudavam na contenção das crianças para realizar diferentes procedimentos no hospital destacou que eles desempenharam um papel protetor nesse contexto^(32)^. Assim, movidos pelo desejo de que seus filhos se sentissem seguros, confortavam as crianças e apoiavam os profissionais de saúde na execução do procedimento. Levando em conta os papéis que os pais podem assumir durante a assistência, os autores recomendam que os pais sejam incluídos durante a realização de procedimentos de saúde nas crianças.

Com o intuito de amenizar possíveis danos à criança, a equipe de enfermagem participante deste estudo destacou que tinha como prática, após a conclusão do procedimento de saúde com o uso da contenção, ofertar alguma estratégia para controlar os possíveis danos, sendo o colo do familiar e o reforço positivo as mais frequentes. Na prática assistencial, parece haver esforços dos profissionais de saúde para empregar, além da contenção, outras estratégias que facilitem a execução de procedimentos de saúde na pediatria. Nessa perspectiva, pesquisa realizada na Noruega com o objetivo de descrever como enfermeiros e médicos lidavam com a resistência de crianças pré-escolares durante a punção venosa periférica mostrou a utilização de diferentes estratégias^(28)^. Dentre essas, foram identificados comentários para reconhecer as emoções e angústias da criança e, mais comumente, para distraí-la, por meio de perguntas e elogios que desviavam a sua atenção do procedimento técnico^(28)^.

Por outro lado, pesquisa desenvolvida com 712 profissionais de saúde que atuavam em dez países diferentes revelou que estes buscavam considerar as escolhas das crianças; entretanto, as inúmeras demandas dos serviços frequentemente levavam ao uso da contenção visando concluir o procedimento de forma mais rápida^(33)^. Além disso, os participantes expressaram dificuldades para acalmar e envolver a criança quando outras estratégias não funcionavam, enquanto o procedimento precisava ser concluído, o que se tornou um dilema clínico a ser gerenciado^(33)^.

Outro resultado frisado na presente investigação tem a ver com o registro sobre a contenção no prontuário da criança. Quanto a isso, os dados mostraram que os profissionais registravam o procedimento, bem como o que exigiu o uso da contenção, a presença e a autorização do familiar e/ou responsável. Todavia, o consentimento da criança foi a informação menos registrada no cotidiano de trabalho. Nesse sentido, pesquisa realizada em um hospital infantil da Inglaterra sublinhou, com base na observação não participante de 31 procedimentos de saúde realizados em crianças e de entrevistas com os profissionais de saúde, incluindo enfermeiros, que o registro de informações sobre a contenção não era realizado rotineiramente pelos profissionais no prontuário da criança^(5)^. Todavia, de acordo com as boas práticas recomendadas pela *iSupport collaboration*, que propõe uma abordagem que priorize os direitos das crianças e minimize os possíveis danos decorrentes da contenção, essa documentação é um dos elementos-chave^(1,26,27)^.

É notório na literatura científica que a prática de contenção da criança impõe dilemas éticos e morais para os profissionais de saúde^(1,2,3,6,28)^. Por isso, torna-se indispensável o desenvolvimento de novas investigações com a perspectiva de conhecer como os profissionais de saúde respondem a esse fenômeno na assistência clínica, visando assim torná-los conscientes das próprias práticas, além de permitir que reflitam sobre o uso de estratégias que respeitem os direitos das crianças, mesmo com o desafio que se impõe diante da resistência infantil a procedimentos de saúde^(28)^.

Por fim, deve-se considerar que os profissionais de saúde, incluindo a equipe de enfermagem, estão inseridos em um contexto formativo, assistencial, gerencial e de saúde que pode impor restrições significativas no atendimento das necessidades específicas da criança e sua família. Assim, salienta-se que a prática de conter a criança durante procedimentos de saúde parece ser afetada diretamente pela formação e capacitação/treinamento recebidos pelos profissionais^(20)^. Esses são aspectos importantes a serem levados em conta na reflexão e fundamentação dos avanços necessários para garantir os direitos assegurados às crianças e, consequentemente, a fim de que prevaleça um cuidado de enfermagem pautado em princípios éticos.

Os resultados deste estudo estão sujeitos a limitações. Salienta-se que as descobertas aqui relatadas dizem respeito à experiência dos profissionais de enfermagem atuantes no sistema de saúde do Brasil e, portando, limitam-se a essa realidade assistencial. Além disso, há de se considerar que o nosso país estende-se por dimensões continentais que podem influenciar até mesmo as práticas de enfermagem. Da mesma forma, cabe ressaltar que, apesar dos esforços dos pesquisadores, não foi alcançada a amostra proporcional na macrorregião Nordeste, nos estratos de enfermeiros(as) e técnicos(as)/auxiliares de enfermagem, e na macrorregião Sul no estrato de técnicos(as)/auxiliar de enfermagem, o que pode de alguma maneira interferir nas interpretações dos achados. O tipo de amostra por conveniência e o viés de memória também devem ser considerados, uma vez que os profissionais de enfermagem formados há muito tempo podem não se lembrar de experiências prévias de forma acurada.

## CONCLUSÃO

O método de contenção configurou-se como uma prática comum para a realização pela equipe de enfermagem de procedimentos como punção venosa, administração de medicamentos e sondagens nas crianças hospitalizadas, sendo a contenção física, contenção com apoio e contenção psicológica as técnicas mais empregadas. A sua utilização foi justificada em razão do comportamento da criança, a garantia da sua segurança, sua recusa em cooperar e, ainda, pela faixa etária. Ademais, os profissionais de enfermagem priorizam a família e/ou responsável em detrimento da criança para dar explicação sobre a contenção e a obtenção do consentimento para realizá-la. Depois da realização da contenção, era comum a oferta do colo do familiar e de reforço positivo para amenizar os possíveis danos. No prontuário da criança, os profissionais registravam informações sobre o procedimento de saúde e o que demandou o uso da contenção, bem como a presença e o consentimento da família e/ou responsável. Todavia, ficou evidenciado pelo relato dos profissionais o despreparo da equipe de enfermagem em virtude da ausência de conteúdos sobre o tema no processo formativo e de atualização/capacitação durante a trajetória profissional.

À vista dos resultados, salienta-se que esses poderão servir como base para a elaboração de diretrizes nacionais, normatização profissional pelo conselho de enfermagem e desenvolvimento de protocolos institucionais. Esses documentos deverão estipular recomendações para a equipe de enfermagem sobre os aspectos envolvidos na prática de contenção da criança hospitalizada e os dilemas éticos enfrentados na assistência clínica. Além disso, poderão fomentar a incorporação deste conteúdo e as suas especificidades no processo de ensino e aprendizagem durante a formação em enfermagem no país, tanto no nível técnico quanto na graduação e pós-graduação.

Por fim, é fundamental destacar a necessidade de ampliar o alcance deste objeto de pesquisa no Brasil sob diferentes pontos de vista. Por isso, instiga-se a realização de mais investigações científicas que contemplem a contenção em diversos contextos assistenciais, além de assegurar o protagonismo da criança. Isso permitirá que o fenômeno seja mapeado de forma abrangente, diversa e nas mais variadas peculiaridades, com o propósito de avançar as práticas de enfermagem com respeito e consideração às emoções e os direitos das crianças.

## Data Availability

Todo o conjunto de dados que dá suporte aos resultados deste estudo foi publicado no próprio artigo.

# Translation: Restricción de niños hospitalizados para procedimientos: prácticas de profesionales de enfermería en Brasil^*^

**DOI:** 10.1590/1980-220X-REEUSP-2025-0453es

**Authors:** Ronaldo Antonio da Silva, Maria Aparecida Munhoz Gaíva

**Affiliations:** 1 Universidade do Estado de Mato Grosso, Diamantino, MT, Brasil.; 2 Universidade Federal de Mato Grosso, Cuiabá, MT, Brasil.

**Keywords:** Niño Hospitalizado, Vías Clínicas, Restricción Física, Grupo de Enfermería, Atención de Enfermería

## Abstract

**Objetivo:**

Describir las prácticas de restricción empleadas por los profesionales de enfermería brasileños al realizar procedimientos sanitarios en niños hospitalizados.

**Método:**

Se realizó un estudio transversal con 897 profesionales que trabajan en el cuidado de niños hospitalizados en el país. Los datos se recopilaron entre mayo de 2024 y enero de 2025, utilizando un cuestionario con información sociodemográfica y otro sobre prácticas de contención, que fue sometido a validación de contenido y a pruebas preliminares. El banco fue analizado en *software R.*

**Resultados:**

La restricción se caracterizó como un método común; sin embargo, los profesionales informaron que no recibieron capacitación durante su formación académica o práctica profesional para llevarla a cabo. Las técnicas más utilizadas fueron las físicas, con apoyo, y las psicológicas, en procedimientos como la venopunción, la administración de medicamentos y el cateterismo. La decisión de utilizarla estuvo relacionada con el comportamiento del niño, con la necesidad de garantizar su seguridad y con su negativa a cooperar.

**Conclusión:**

Se describieron las prácticas de restricción utilizadas por el personal de enfermería brasileño para la atención pediátrica, basándose en las técnicas, los motivos, el consentimiento, la participación familiar, el control de los posibles daños y la documentación en los registros médicos.

## INTRODUCCIÓN

La contención se define como cualquier método que limite el movimiento, la actividad física o el acceso normal de una persona a su cuerpo, y ha sido adoptada con frecuencia por los profesionales de la salud en la atención pediátrica^(1,2,3)^. Esta práctica se utiliza durante la hospitalización del niño para diferentes procedimientos de salud, tales como: punción venosa, administración de medicamentos, cateterismos, análisis de sangre y exámenes radiológicos^(1,2,3)^. Sin embargo, la contención del niño está rodeada de incertidumbres y controversias respecto a las implicaciones éticas y morales relacionadas con la actuación profesional, además de los riesgos y consecuencias a corto, mediano y largo plazo en la vida y la salud de los niños que son contenidos^(3,4)^.

Se sabe que la práctica de la contención durante la realización de procedimientos de salud puede tener efectos perjudiciales para el niño, tal como lo señalan las evidencias científicas. Investigadores suecos, al analizar las narrativas de los niños que se sometían a diferentes procedimientos de salud no urgentes, destacaron que, para estos niños, la contención significaba sufrimiento emocional^(4)^. En esta investigación, los niños relataron que se sentían heridos al verse obligados a someterse a tales procedimientos, y que los recuerdos de esos momentos permanecían durante mucho tiempo. Además, según la inferencia de los autores, estos recuerdos negativos pueden afectar la interacción del niño con los profesionales en futuros servicios de salud.

En este sentido, se sabe que la angustia anticipada es una realidad en la vida de los niños hospitalizados y sometidos a procedimientos de salud. Según la literatura, pueden experimentar angustia tanto antes de los procedimientos como al sufrir la contención contra su voluntad^(1,4,5,6)^. Entre los factores que desencadenan y potencian el sentimiento de angustia anticipada antes de un procedimiento de salud se encuentra la experiencia negativa de los niños y sus familiares en procedimientos previos y hospitalizaciones^(7)^.

En cuanto a la aplicación de la contención en la práctica, una reciente revisión de alcance destacó que, entre los profesionales de la salud, los que integran el equipo de enfermería fueron citados en el 61,5 % de los estudios como los involucrados en la contención de los niños^(3)^. Además, se catalogaron estudios realizados en diferentes países, con especial énfasis en los del continente europeo. Sin embargo, no se identificaron estudios publicados sobre este tema en Brasil, lo que indicó un vacío en la literatura científica del país^(3)^. Ante la problemática presentada, se planteó la siguiente pregunta de investigación: ¿cuáles son las prácticas de contención utilizadas por los profesionales de enfermería brasileños para la ejecución de procedimientos de salud en el niño hospitalizado?

Esta investigación tiene el potencial de acercarse a las prácticas cotidianas de los profesionales de enfermería en los servicios de salud de Brasil y, de este modo, comprender cómo se presenta el fenómeno de la contención de niños hospitalizados en este contexto social y cultural, sobre el cual aún hay pocos datos disponibles. Estos resultados contribuirán a la literatura científica internacional, con el fin de ampliar el alcance para respaldar enfoques que prioricen la garantía de los derechos y aseguren el bienestar físico, emocional y psicológico de los niños durante la realización de procedimientos de salud no urgentes. Así, este estudio tuvo como objetivo describir las prácticas de contención empleadas por los profesionales de enfermería brasileños al realizar procedimientos de salud en niños hospitalizados.

## MÉTODO

### Tipo de Estudio

Se trata de un estudio transversal.

### Lugar, Población, Criterios de Selección y Muestra

El lugar del estudio fue Brasil, país dividido en cinco macrorregiones a efectos de organización político-administrativa: Norte, Nordeste, Sur, Sudeste y Centro-Oeste, que abarcan las 27 Unidades de la Federación^(8)^.

La población estuvo compuesta por enfermeros, técnicos y/o auxiliares de enfermería que trabajaban en la atención de niños hospitalizados en las cinco macrorregiones del país. El muestreo se realizó por autoselección, una técnica no probabilística empleada cuando los participantes tienen características relevantes para la investigación y se ofrecen como voluntarios para participar^(9)^. En este estudio, el criterio de inclusión fue contar con una experiencia profesional de al menos seis meses en la atención de enfermería pediátrica y/o neonatológica, tiempo que se consideró adecuado para garantizar la familiaridad y la experiencia con las prácticas de contención del niño hospitalizado, y se excluyó a los profesionales que participaron en la prueba piloto.

En cuanto al tamaño de la muestra, utilizando los datos de la investigación sobre el perfil de la enfermería en Brasil^(10)^, se realizó el cálculo de muestreo proporcional para cada macrorregión brasileña en los estratos de enfermeros/as, técnicos/as y auxiliares de enfermería. Con un nivel de confianza del 95 %, se requería una muestra mínima de 385 sujetos para cada estrato. La muestra obtenida estuvo compuesta por 897 profesionales de enfermería.

### Recolección de Datos

La recolección de datos se llevó a cabo entre mayo de 2024 y enero de 2025. Se utilizaron: un cuestionario con preguntas sobre información sociodemográfica y otro sobre la experiencia con la contención en los procedimientos de salud en el niño durante la hospitalización. Este segundo cuestionario se elaboró a partir de una revisión bibliográfica sobre el tema^(3)^ y se sometió a validación de contenido para lograr legitimidad y credibilidad. Participaron en esta etapa seis especialistas: tres docentes e investigadoras, una enfermera supervisora de pediatría, una enfermera de atención pediátrica y una enfermera de atención en la Unidad de Terapia Intensiva Neonatal (UTIN). Tras la revisión de los dominios y los ítems, este cuestionario presentó un índice de validez de contenido (IVC) de 0,86, lo que se considera un nivel de concordancia satisfactorio^(11,12)^.

Así, se investigaron variables relacionadas con las características sociodemográficas (categoría profesional, edad, sexo, estado civil, presencia de hijos y macrorregión de residencia); formación profesional (haber estudiado o realizado un curso reciente sobre contención, poseer o cursar un posgrado en el área pediátrica/neonatológica y la modalidad); práctica profesional (área y tiempo de trabajo); y prácticas de contención (uso, quién las realiza, técnica, procedimiento asociado, motivos, percepción de la necesidad por edad/sexo, comunicación/consentimiento con el familiar y el niño, presencia/ayuda del familiar, control de daños y registro en la historia clínica, incluyendo el tipo de información registrada).

Tras este proceso, los cuestionarios se registraron en *Google Forms* ^®^ y se sometieron a una prueba piloto, aplicándose a nueve profesionales de enfermería que trabajaban en unidades de atención de urgencias que ofrecían asistencia de urgencia y emergencia a recién nacidos, neonatos y/o niños en el estado de Mato Grosso, macrorregión Centro-Oeste. Posteriormente, el *enlace* con los cuestionarios y el Formulario de Consentimiento Libre e Informado se enviaron a los participantes por *correo electrónico*, redes sociales (*Instagram* ^®^ y/o *Facebook* ^®^) y *WhatsApp* ^®^. Además, el *enlace* se compartió en las cinco macrorregiones del país con el apoyo de las asociaciones profesionales, instituciones educativas, grupos de investigación y hospitales con atención pediátrica.

### Análisis y Tratamiento de los Datos

La base de datos se generó en una hoja de cálculo de Microsoft Excel^®^, que fue revisada por los investigadores para garantizar su consistencia e integridad. Además, se renombraron las columnas para facilitar el procesamiento en el software de análisis. Posteriormente, la base de datos se analizó en el software R^(13)^, calculando las frecuencias absolutas y relativas para las variables cualitativas y cuantitativas, así como las medidas de tendencia central y dispersión, incluyendo la media, la desviación estándar y los valores mínimos y máximos.

### Aspectos Éticos

Esta investigación respetó los preceptos éticos de la investigación con seres humanos en Brasil y fue aprobada por el Comité de Ética en Investigación de la Universidad Federal de Mato Grosso (CEP-SAÚDE/UFMT) bajo el dictamen n.^o^ 6.672.454/2024 y CAAE n.^o^ 76648023.4.0000.8124, de conformidad con las resoluciones n.^o^ 466/2012 y 510/2016 del Consejo Nacional de Salud^(14,15)^. Además, se siguieron las directrices establecidas en la circular n.^o^ 2/2021 de la Comisión Nacional de Ética en Investigación (CONEP), que orienta los procedimientos en investigaciones con cualquier etapa en un entorno virtual en el país^(16)^.

## RESULTADOS

Participaron en este estudio 897 profesionales de enfermería, cuyas características sociodemográficas, de formación y ámbito de actuación se presentan en la tabla 1. La mayor proporción de los profesionales residía en la macrorregión Sudeste (n = 558; 62,21 %), seguida de la región Centro-Oeste (n = 113; 12,60 %), Sur (n = 91; 10,14 %), Nordeste (n = 68; 7,58 %) y Norte (n = 67; 7,47 %). En cuanto a la categoría profesional, la mayoría eran enfermeros/as (n = 462; 51,51 %), seguidos por técnicos/as de enfermería (n = 349; 38,91 %) y auxiliares de enfermería (n = 86; 9,59 %). La edad de los participantes osciló entre los 18 y los 72 años, con una media de 40,9 años y una desviación estándar de 10,12 años, lo que indica un rango de edad diversificado. La gran mayoría de los encuestados eran mujeres (n = 785; 87,51 %) y, en cuanto al estado civil, la mayor parte se declaró casada (n = 472; 52,62 %), y en lo que respecta a la presencia de hijos, 627 profesionales (69,90 %) afirmaron tener hijos.

**Tabla 1:** Características sociodemográficas, de formación y de ejercicio profesional del personal de enfermería objeto del estudio – Cuiabá, MT, Brasil, 2025.

Variables	n (%)	Media (DE)
Categoría profesional		
Enfermero/a	462 (51,51 %)	
Técnico/a de enfermería	349 (38,91 %)	
Auxiliar de enfermería	86 (9,59 %)	
Edad (años)		40,87 (10,12)
Sexo		
Masculino	112 (12,49 %)	
Femenino	785 (87,51 %)	
Estado civil		
Soltero/a	326 (36,34 %)	
Casado(a)	472 (52,62 %)	
Divorciado(a)	90 (10,03 %)	
Viudo/a	9 (1,0 %)	
Tenía hijos		
Sí	627 (69,90 %)	
No	270 (30,10 %)	
Macrorregión de residencia		
Norte	67 (7,47 %)	
Noreste	68 (7,58 %)	
Centro-Oeste	113 (12,60 %)	
Sudeste	558 (62,21 %)	
Sur	91 (10,14 %)	
Estudió contenidos sobre contención durante la formación		
Sí	285 (31,77 %)	
No	612 (68,23 %)	
Ha realizado un curso de formación o actualización sobre contención en los últimos 12 meses		
Sí	81 (9,03 %)	
No	816 (90,97 %)	
Tenía un posgrado en el área pediátrica/neonatológica		
Sí	227 (25,31 %)	
No	670 (74,69 %)	
Modalidad de la formación de posgrado en el área		
Residencia	22 (9,69 %)	
Especialización	150 (66,08 %)	
Máster profesional	9 (3,96 %)	
Máster académico	19 (8,37 %)	
Doctorado	20 (8,81 %)	
Postdoctorado	7 (3,08 %)	
Estaba cursando estudios de posgrado en el área		
Sí	67 (7,47 %)	
No	830 (92,53 %)	
Modalidad de los estudios de posgrado que cursaba		
Residencia	10 (14,93 %)	
Especialización	35 (52,24 %)	
Máster profesional	1 (1,49 %)	
Máster académico	6 (8,96 %)	
Doctorado	14 (20,90 %)	
Postdoctorado	1 (1,49 %)	
Área de actividad profesional^*^		
Salud del recién nacido	408 (45,48 %)	
Salud infantil	588 (65,55 %)	
Salud del adolescente	491 (54,74 %)	
Antigüedad laboral (años)		7,2 (8,69)

^*^El participante podía marcar más de una opción; DP: Desviación estándar.

En cuanto a la formación, 612 (68,23 %) profesionales informaron que no se les había ofrecido ningún contenido relativo a la contención del niño durante la licenciatura o el curso técnico/auxiliar de enfermería. En lo que respecta a la educación continua, 816 (90,97 %) afirmaron no haber participado en ningún curso de capacitación o actualización sobre contención de niños en los últimos 12 meses. Aún sobre la formación, 227 profesionales (25,31 %) indicaron poseer algún tipo de posgrado en el área pediátrica o neonatológica, siendo la especialización la modalidad más recurrente (n = 150; 66,08 %), seguida de la residencia (n = 22; 9,69 %), el doctorado (n = 20; 8,81 %), el máster académico (n = 19; 8,37 %), el máster profesional (n = 9; 3,96 %) y el posdoctorado (n = 7; 3,08 %). En el momento de la recolección, 67 profesionales (7,47 %) estaban cursando algún posgrado en estas áreas; la especialización representó nuevamente la modalidad principal (n = 35; 52,24 %), seguida del doctorado (n = 14; 20,9 %) y la residencia (n = 10; 14,93 %).

En cuanto al ámbito de actuación profesional, los participantes pudieron señalar múltiples áreas y la mayoría trabajaba en salud infantil (n = 588; 65,55 %), seguida de aquellos que trabajaban en salud adolescente (n = 491; 54,74 %) y en salud neonatal (n = 408; 45,48 %). En cuanto al tiempo de trabajo en enfermería pediátrica y/o neonatológica, los datos indicaron una variación entre 1 y 50 años de experiencia (7,2 ± 8,69).

La tabla 2 destaca las prácticas de inmovilización empleadas por el personal de enfermería brasileño en los procedimientos sanitarios realizados a niños hospitalizados. La mayoría del personal de enfermería (n = 760; 80,73 %) afirmó haber utilizado alguna vez el método de inmovilización para realizar procedimientos sanitarios a niños hospitalizados, lo que pone de manifiesto una amplia adopción de esta práctica en el contexto hospitalario. Se constató, además, que los técnicos y técnicas de enfermería (n = 647; 72,13 %) fueron señalados como los que más realizaban la contención en el entorno laboral.

**Tabla 2:** Prácticas de contención utilizadas por el personal de enfermería brasileño para realizar procedimientos sanitarios en niños hospitalizados – Cuiabá, MT, Brasil, 2025.

Variables	n (%)
Utilizó la contención	
Sí	760 (84,73 %)
No	137 (15,27 %)
Profesional de enfermería que más realiza la contención en su lugar de trabajo	
Enfermero/a	175 (19,51 %)
Técnico/a de enfermería	647 (72,13 %)
Auxiliar de enfermería	75 (8,36 %)
Técnica utilizada en su servicio^*^	
Física	
Sí	590 (65,77 %)
No	307 (34,23 %)
Mecánica	
Sí	257 (28,65 %)
No	640 (71,35 %)
Química	
Sí	202 (22,52 %)
No	695 (77,48 %)
Psicología	
Sí	349 (38,91 %)
No	548 (61,09 %)
Contención con apoyo	
Sí	441 (49,16 %)
No	456 (50,84 %)
Procedimiento sanitario^*^	
Administración de medicamentos	
Sí	448 (49,94 %)
No	449 (50,06 %)
Vendaje	
Sí	272 (30,32 %)
No	625 (69,68 %)
Vacunación	
Sí	255 (28,43 %)
No	642 (71,57 %)
Punción venosa	
Sí	795 (88,63 %)
No	102 (11,37 %)
Encuestas	
No	384 (42,81 %)
Sí	513 (57,19 %)
Motivo(s) para utilizar la contención^*^	
Edad	
Sí	343 (38,24 %)
No	554 (61,76 %)
Comportamiento (agitada/agresiva)	
Sí	731 (81,49 %)
No	166 (18,51 %)
Negativa a cooperar	
Sí	476 (53,08 %)
No	421 (46,93 %)
Presencia de alguna discapacidad	
Sí	228 (25,42 %)
No	669 (74,58 %)
Capacidad de comprensión	
Sí	429 (47,83 %)
No	468 (52,17 %)
Garantizar la seguridad del niño	
Sí	720 (80,27 %)
No	177 (19,73 %)
Necesidad de completar el procedimiento	
Sí	460 (51,28 %)
No	437 (48,72 %)
Tiempo limitado para realizar el procedimiento	
Sí	192 (21,40 %)
No	705 (78,60 %)
Restricción por grupo de edad	
Sí	803 (89,52 %)
No	94 (10,48 %)
Contención por sexo	
Sí	268 (29,88 %)
No	629 (70,12 %)
Informaba al familiar y/o responsable	
Sí	814 (90,75 %)
No	31 (3,46 %)
A veces	52 (5,8 %)
Se lo decía al niño	
Sí	568 (63,32 %)
No	105 (11,71 %)
A veces	224 (24,97 %)
Solicitaba el consentimiento del familiar y/o del responsable	
Sí	617 (68,78 %)
No	191 (21,29 %)
A veces	89 (9,92 %)
Solicitaba el consentimiento del niño	
Sí	304 (33,89 %)
No	398 (44,37 %)
A veces	195 (21,74 %)
El familiar está presente en el momento de la contención	
Sí	757 (84,39 %)
No	24 (2,68 %)
A veces	116 (12,93 %)
Se orienta al familiar para que ayude a sujetar físicamente al niño	
Sí	648 (76,25 %)
No	64 (7,13 %)
A veces	149 (16,61 %)
Control de posibles daños	
Sí	464 (51,73 %)
No	227 (25,31 %)
A veces	206 (22,97 %)
Estrategias de control^*^	
Abrazo del familiar	
Sí	392 (43,70 %)
No	505 (56,30 %)
Pomada para prevenir hematomas	
Sí	51 (5,69 %)
No	846 (94,31 %)
Refuerzo positivo (certificados y regalo)	
Sí	175 (19,51 %)
No	722 (80,49 %)
Otros	
Sí	56 (6,24 %)
No	841 (93,76 %)
Lo anotaba en la historia clínica	
Sí	630 (70,23 %)
No	161 (17,95 %)
A veces	106 (11,82 %)
Información registrada^*^	
Procedimiento y motivo por el que se requirió el uso de la inmovilización	
Sí	494 (55,07 %)
No	403 (44,93 %)
Tipo de sujeción utilizada	
Sí	248 (27,65 %)
No	649 (72,35 %)
Profesional sanitario que ayudó a sujetar al niño	
Sí	153 (17,06 %)
No	744 (82,94 %)
Presencia del familiar/responsable	
Sí	262 (29,21 %)
No	635 (70,79 %)
Consentimiento del familiar/responsable	
Sí	262 (29,21 %)
No	635 (70,79 %)
Consentimiento del niño	
Sí	87 (9,70 %)
No	810 (90,30 %)
Aspectos que influyeron en la decisión de contener	
Sí	185 (20,62 %)
No	712 (79,38 %)

^*^El participante podía marcar más de una opción.

En cuanto a la técnica de contención utilizada por los profesionales, la contención física fue la más frecuente (n = 590; 65,77 %), seguida de la contención con apoyo (n = 441; 49,16 %) y la contención psicológica (n = 349; 38,91 %). En cuanto a los procedimientos sanitarios en los que los profesionales de enfermería utilizaban con mayor frecuencia la contención, destacaron: la punción venosa (n = 795; 88,63 %), la administración de medicamentos (n = 448; 49,94 %) y las sondas (n = 384; 42,81 %).

Los motivos mencionados por los profesionales de enfermería para el uso de la contención en el niño hospitalizado fueron: el comportamiento del niño (n = 731; 81,49 %), garantizar su seguridad (n = 720; 80,27 %) y, además, su negativa a cooperar (n = 476; 53,08 %). En cuanto a la necesidad de la contención, la mayoría de los profesionales (n = 629; 70,12 %) no identificó diferencias en función del sexo de los niños.

Al analizar los datos sobre la explicación del tipo de contención adoptada, se observó que la mayor proporción de profesionales de enfermería consideraba informar a la familia y/o al responsable (n = 814; 90,75 %) en detrimento del niño (n = 568; 63,32 %). Además, en lo que respecta a la solicitud de consentimiento para llevar a cabo la contención, ya sea verbal o por escrito, los profesionales consideraban obtener el consentimiento de la familia y/o del responsable (n = 617; 68,78 %), pero 398 (44,37 %) informaron de que no solicitaban autorización al niño.

En cuanto a la participación de la familia, los profesionales de enfermería destacaron que esta permanecía presente en el momento de la contención (n = 757; 84,39 %), y que con frecuencia se le indicaba que ayudara en la contención física del niño (n = 648; 76,25 %).

La mayoría de los profesionales de enfermería (n = 464; 51,73 %) ofrecía alguna estrategia para controlar posibles daños una vez concluido el procedimiento sanitario en el que se había producido la contención. Entre las estrategias más citadas para minimizar los posibles daños se encuentran el regazo del familiar (n = 392; 43,70 %) y el refuerzo positivo (n = 175; 19,51 %).

La mayoría de los participantes afirmó que registraba la información sobre la contención en la historia clínica del niño (n = 630; 70,23 %). Y la información sobre el procedimiento y lo que exigía el uso de la contención fue la que se registró con mayor frecuencia (n = 494; 55,07); sin embargo, el registro en la historia clínica de la obtención del consentimiento del niño era menos común (n = 87; 9,70 %).

## DISCUSIÓN

La distribución de los profesionales de enfermería que participaron en la investigación se concentró principalmente en la macrorregión Sudeste, mientras que el menor número de participantes se concentró en las macrorregiones Norte y Nordeste. Según datos publicados por el Consejo Federal de Enfermería (COFEN)^(17)^, el estado de São Paulo cuenta con el mayor número de profesionales de enfermería (664 956), seguido de Río de Janeiro (361 886) y Minas Gerais (262 394), todos ellos ubicados en el Sudeste. El único estudio brasileño que aborda el perfil de los profesionales de enfermería que trabajan en el área pediátrica y neonatal mostró que el lugar de residencia de los enfermeros pediatras y neonatólogos más citado se encontraba en las macrorregiones Sudeste y Sur^(18)^.

En este estudio, se observó una amplia adopción del método de contención para la realización de procedimientos de salud en niños hospitalizados por parte de los profesionales de enfermería brasileños, sobre todo por los técnicos y técnicas de enfermería. La variación en su uso en el entorno laboral entre los miembros del equipo de enfermería puede estar relacionada con las atribuciones de cada categoría profesional, respaldadas desde 1986 por la Ley n.º 7.498, que regula el ejercicio de la enfermería en Brasil^(19)^. En ella se establece que los profesionales de enfermería ejerzan todas las actividades de enfermería, sumadas a algunas funciones que les son exclusivas, mientras que los técnicos y auxiliares de enfermería ejerzan, entre otras atribuciones, la ejecución de las acciones asistenciales de enfermería, excepto las exclusivas del enfermero^(19)^. En consecuencia, al estar más presentes en la atención directa a los pacientes pediátricos y ser numéricamente más numerosos que los enfermeros, estos profesionales pueden estar más sujetos al uso de la contención.

Corroborando los hallazgos sobre el uso frecuente de la contención por parte del personal de enfermería en la atención pediátrica brasileña, también se observó esta evidencia en otros estudios realizados a nivel internacional^(2,20,21)^. En Irlanda, los investigadores analizaron la percepción de 50 enfermeras de un hospital infantil sobre el uso de la contención, y los hallazgos revelaron que el 76 % de ellas utilizó el método en niños durante alguna atención clínica^(21)^. En otro contexto, 30 profesionales de la salud, entre ellos enfermeras, que trabajaban en cuatro hospitales con instalaciones pediátricas en Francia, también informaron que recurrir a la contención para el cuidado del niño era una práctica común^(2)^. Y, por último, en una investigación internacional que analizó la perspectiva de los profesionales de la salud sobre las prácticas de contención infantil, en la que el 75 % eran enfermeros, se puso de manifiesto que se trataba de una práctica muy frecuente entre los profesionales que trabajaban en Australia, Nueva Zelanda y el Reino Unido^(20)^.

En lo que respecta a la formación y la cualificación profesional, el equipo de enfermería participante informó no haber recibido contenidos preparatorios sobre la contención en el proceso formativo y, además, no haber recibido actualización o capacitación sobre el tema durante su vida profesional. Este preocupante resultado también se identificó en investigaciones realizadas en otros países, como Irlanda, donde el 98,5 % de las enfermeras encuestadas informaron no haber recibido capacitación sobre la contención de niños en el hospital infantil en el que trabajaban^(21)^.

En este contexto, una investigación dirigida por una enfermera del Reino Unido obtuvo resultados que enriquecen el debate sobre la importancia de la educación continua, ya que mostró que el 60 % de los profesionales de la salud participantes en la investigación también informaron no haber recibido capacitación sobre este tema^(20)^. Para quienes sí recibieron capacitación, esta fue puntual y se llevó a cabo durante la licenciatura, la posgrado o en cursos en el entorno laboral^(20)^. Además, se observó una asociación estadísticamente significativa en la frecuencia del uso de la contención entre los profesionales capacitados y los no capacitados, siendo que los no capacitados la utilizaban con mayor frecuencia^(20)^.

La inversión en la educación continua del profesional es una estrategia para garantizar una atención segura y libre de daños al paciente. En lo que respecta a la contención, a pesar de ser un método ampliamente adoptado por los profesionales de la salud en la atención infantil, se sabe o se discute poco sobre sus repercusiones para el niño sometido a este procedimiento^(4)^. Teniendo en cuenta el potencial que tiene la contención de causar sufrimiento emocional al niño, los profesionales de la salud deben estar capacitados para implementar, en sus lugares de trabajo, alternativas que promuevan experiencias positivas y respeten los derechos y el bienestar de los niños^(1,2,3)^.

Específicamente en lo que respecta a los derechos, en Brasil, el Consejo Nacional de los Derechos del Niño y del Adolescente (CONANDA), a partir de la Resolución n.^o^ 41/1995, estableció los derechos de los niños y adolescentes hospitalizados^(22)^. De este modo, se garantiza al niño el derecho a tener un conocimiento adecuado sobre su enfermedad, los cuidados terapéuticos que se le prestarán, los diagnósticos y pronósticos, respetando su fase cognitiva, a recibir apoyo psicológico cuando sea necesario y, además, a la protección integral de su dignidad física, psíquica y moral^(22)^.

Cabe destacar que, en la realidad brasileña, aún no contamos con ninguna normativa o directriz específica sobre el uso de la contención en el niño. La única normativa que aborda el tema de la contención en el país fue propuesta en 2012 por el COFEN, mediante la resolución n.^o^ 427/2012, que trataba específicamente sobre la contención mecánica y los procedimientos de enfermería en el empleo de este tipo de contención^(23)^. Dicha resolución fue derogada con la publicación de una nueva, la resolución n.^o^ 746, en marzo de 2024^(24)^. En esta actualización de la normativa profesional, se eliminó la salvedad, descrita en el segundo inciso del artículo 4 de la antigua resolución, que se refería a la necesidad de un mayor rigor en el monitoreo de la contención mecánica en niños y adolescentes. A este respecto, el artículo 2 de la nueva resolución destaca la necesidad de un monitoreo general, para promover la seguridad del paciente y prevenir daños y eventos adversos.

Para debatir las cuestiones relacionadas con el uso de la contención en la infancia, se llevó a cabo una revisión de alcance con el objetivo de mapear intervenciones o directrices para prevenir el uso de la contención durante los procedimientos de salud en niños hospitalizados. En este estudio, se identificaron 11 directrices, políticas o recomendaciones que tenían este enfoque y, de ellas, solo una era específica sobre la contención y estaba dirigida a los profesionales de la salud^(25)^. Entre sus recomendaciones, se destacó la importancia de ofrecer capacitación, evaluación y documentación, para que los profesionales adquieran conocimientos y desarrollen habilidades para abordar esta cuestión en la práctica clínica^(25)^. Y, por último, los autores destacaron también la necesidad de establecer directrices relativas al uso de la contención en pediatría, con la inclusión de definiciones, el establecimiento de los derechos de los niños, así como las responsabilidades de los profesionales de la salud en estas situaciones.

Con el fin de garantizar los derechos de los niños en su relación con los servicios de salud, con el objetivo de contribuir a la reducción de daños durante la realización de procedimientos de salud, la *iSupport collaboration* propuso las «Buenas prácticas basadas en los derechos de los niños sometidos a procedimientos de salud», definiendo acciones que orientan a los profesionales de la salud en el uso de la contención con apoyo durante la realización de procedimientos de salud para garantizar, de este modo, el respeto de los derechos de los niños^(1,26,27)^.

En lo que respecta al uso de la contención, según la experiencia de los profesionales de enfermería participantes en este estudio, la técnica de contención más empleada fue la física y el procedimiento de salud que más exigió su uso fue la punción venosa. Entre los motivos que justificaron su uso, se evidenciaron algunos relacionados con el niño, como su comportamiento, la necesidad de garantizar su seguridad, su negativa a cooperar para la realización del procedimiento y, además, dependiendo del grupo de edad, la necesidad de contención es mayor. Otras investigaciones corroboran estos hallazgos y destacan que, entre los factores señalados por los profesionales de la salud como capaces de influir en la necesidad de contener a un niño para la realización de procedimientos de salud, se destacaron la seguridad del niño (31 %), la edad (31 %) y la angustia (9 %)^(20)^. A su vez, según la experiencia de enfermeros y médicos que trabajaban en la unidad infantil de un hospital noruego, las técnicas de contención física y química fueron las más empleadas durante la punción venosa periférica de niños en edad preescolar^(28)^.

La literatura señala de manera consistente que el proceso de hospitalización puede acarrear impactos negativos para el desarrollo emocional del niño^(1,4,29)^. Esto se debe a que, durante ese período, el niño es retirado de su entorno familiar y comienza a experimentar situaciones desconocidas que con frecuencia implican dolor como consecuencia de los procedimientos de salud^(1,29)^. En consecuencia, esta experiencia se interpreta como estresante y genera sentimientos negativos que pueden repercutir a lo largo de toda su vida^(4,29)^.

Así, con el fin de minimizar los sentimientos de este tipo desencadenados por la hospitalización del niño, se han probado nuevas tecnologías en Brasil para ayudar en los procedimientos de salud. Desde esta perspectiva, en un hospital público infantil del sur de Brasil se utilizaron gafas de realidad virtual durante la inserción de catéteres intravenosos periféricos en emergencias pediátricas, y demostraron ser una estrategia prometedora^(30)^. Los niños y los familiares que participaron en la investigación evaluaron el uso de la realidad virtual como un método positivo para reducir los sentimientos negativos vinculados a la realización del cateterismo. Además, los autores destacaron que el 91,6 % de los niños no mostraron resistencia verbal ni se resistieron durante el procedimiento, y el 66,8 % no necesitaron ser inmovilizados^(30)^.

Otro resultado que se destacó en el presente estudio se refiere a la información sobre la inmovilización y la obtención del consentimiento para llevarla a cabo, tanto del familiar y/o responsable como del niño. Al respecto, los datos mostraron que, en la práctica, los profesionales de enfermería informaban sobre la sujeción y obtenían el consentimiento con mayor frecuencia de la familia y/o del responsable que del niño. Además, en Australia, Nueva Zelanda y el Reino Unido, el 76 % de los profesionales de la salud participantes en una investigación informaron solicitar el consentimiento de la familia y/o del responsable antes de sujetar al niño. Sin embargo, en cuanto al consentimiento del propio niño, la mayoría de los participantes informó que esta práctica no era necesaria (33 %) o que no sabía qué responder (29 %)^(20)^.

Teniendo en cuenta los hallazgos de este estudio sobre la obtención del consentimiento del niño, cabe aquí una breve reflexión. Entendemos que el consentimiento del niño debe solicitarse siempre que este tenga capacidad cognitiva para comprender. En el caso de un niño hospitalizado en la UCIN, el profesional debe solicitar el consentimiento al familiar responsable y seguir las recomendaciones de la contención con apoyo, que establecen actitudes de respeto a los derechos de los niños^(1,26,27)^, tales como: discusión entre el equipo sobre la necesidad y la técnica de contención a adoptar; establecer un vínculo con el niño y su familia; durante la contención, velar por la comodidad del niño; garantizar su bienestar tras la finalización del procedimiento; y documentar en el expediente médico la información sobre la contención.

En cuanto a la consideración hacia el niño durante la atención, los autores de una investigación realizada en Francia demostraron que, al enfrentarse a un procedimiento de salud que debe realizarse, el profesional de la salud pierde temporalmente esa consideración^(2)^. Antes, el sujeto del cuidado era el niño; a continuación, pasa a serlo el procedimiento técnico, denominando a este proceso «ceguera empática transitoria»^(2)^.

En este sentido, recientemente, dos revisiones de alcance publicadas sobre el fenómeno de la contención pediátrica, con el objetivo de mapear de manera exhaustiva el conocimiento científico, evidenciaron que los intereses de los niños parecían no tener prioridad en la mayoría de las investigaciones analizadas por los autores^(3,25)^. En ambos casos, el enfoque en las vivencias de los niños con la contención era limitado, ya que la mayor parte de los estudios tenía como objetivo investigar las experiencias y prácticas de los profesionales de la salud y/o de la familia^(3,25)^. Además, muchas investigaciones no incluían información sobre el consentimiento del niño para la contención en la práctica asistencial^(3)^. Por lo tanto, se puede inferir que, además de que los niños ocupan menos espacio en las discusiones académicas y científicas sobre el derecho a estar informados y a dar su consentimiento a las conductas, en la práctica clínica esto no parece ser diferente^(25)^.

Aún sobre este aspecto, una investigación corrobora las reflexiones sobre los resultados obtenidos en la presente investigación, ya que analizó la autonomía garantizada a la niña en la toma de decisiones sobre su atención en salud a la luz del discurso normativo de protección de la infancia en Brasil^(31)^. Los resultados señalaron que la participación del niño en este escenario es un derecho fundamental, pero el tema sigue siendo controvertido, ya que el reconocimiento del derecho a la dignidad y la libertad, la información y la protección depende del juicio de los adultos sobre su capacidad de discernimiento. Proteger al niño es una responsabilidad del Estado, de la sociedad y de la familia^(31)^.

En cuanto a la participación de la familia durante la aplicación de la contención del niño, los resultados de esta investigación destacaron que la familia estaba presente en el momento de la contención y, además, con frecuencia se le indicaba que ayudara en la contención física del niño. A este respecto, la literatura científica concuerda con estos hallazgos, ya que en el 46,7 % de los artículos analizados en una revisión de alcance sobre la contención, se informó de la presencia de los padres o se observó que estos ayudaban a los profesionales de la salud a contener a sus hijos, así como en el 63,3 % de los trabajos estos estaban presentes durante la contención de los niños^(3)^.

En este sentido, una investigación realizada recientemente en Australia con el objetivo de explorar las experiencias de los padres que ayudaban a sujetar a los niños para realizar diferentes procedimientos en el hospital destacó que estos desempeñaban un papel protector en ese contexto^(32)^. Así, movidos por el deseo de que sus hijos se sintieran seguros, consolaban a los niños y apoyaban a los profesionales de la salud en la ejecución del procedimiento. Teniendo en cuenta los roles que los padres pueden asumir durante la atención, los autores recomiendan que se incluya a los padres durante la realización de procedimientos de salud en los niños.

Con el fin de mitigar posibles daños al niño, el equipo de enfermería participante en este estudio destacó que tenía como práctica, tras la conclusión del procedimiento de salud con el uso de la contención, ofrecer alguna estrategia para controlar los posibles daños, siendo el regazo del familiar y el refuerzo positivo las más frecuentes. En la práctica asistencial, parece haber esfuerzos por parte de los profesionales de la salud para emplear, además de la contención, otras estrategias que faciliten la ejecución de procedimientos de salud en pediatría. En esta perspectiva, una investigación realizada en Noruega con el objetivo de describir cómo enfermeros y médicos manejaban la resistencia de niños en edad preescolar durante la punción venosa periférica mostró el uso de diferentes estrategias^(28)^. Entre ellas, se identificaron comentarios para reconocer las emociones y angustias del niño y, más comúnmente, para distraerlo, mediante preguntas y elogios que desviaban su atención del procedimiento técnico^(28)^.

Por otro lado, una investigación realizada con 712 profesionales de la salud que trabajaban en diez países diferentes reveló que estos buscaban tener en cuenta las elecciones de los niños; sin embargo, las innumerables exigencias de los servicios a menudo llevaban al uso de la contención con el fin de concluir el procedimiento de manera más rápida^(33)^. Además, los participantes expresaron dificultades para calmar e involucrar al niño cuando otras estrategias no funcionaban, mientras que el procedimiento debía concluirse, lo que se convirtió en un dilema clínico que había que gestionar^(33)^.

Otro resultado destacado en la presente investigación tiene que ver con el registro de la contención en el expediente médico del niño. Al respecto, los datos mostraron que los profesionales registraban el procedimiento, así como lo que exigió el uso de la contención, la presencia y la autorización del familiar y/o responsable. Sin embargo, el consentimiento del niño fue la información menos registrada en el día a día del trabajo. En este sentido, una investigación realizada en un hospital infantil de Inglaterra subrayó, basándose en la observación no participante de 31 procedimientos de salud realizados en niños y en entrevistas con los profesionales de la salud, incluyendo enfermeros, que el registro de información sobre la contención no era realizado de manera rutinaria por los profesionales en el expediente médico del niño^(5)^. Sin embargo, de acuerdo con las buenas prácticas recomendadas por la *iSupport collaboration*, que propone un enfoque que priorice los derechos de los niños y minimice los posibles daños derivados de la contención, esta documentación es uno de los elementos clave^(1,26,27)^.

Es notorio en la literatura científica que la práctica de la contención del niño plantea dilemas éticos y morales para los profesionales de la salud^(1,2,3,6,28)^. Por ello, resulta indispensable desarrollar nuevas investigaciones con el objetivo de conocer cómo responden los profesionales de la salud a este fenómeno en la atención clínica, con el fin de hacerlos conscientes de sus propias prácticas, además de permitirles reflexionar sobre el uso de estrategias que respeten los derechos de los niños, incluso ante el desafío que supone la resistencia infantil a los procedimientos de salud^(28)^.

Por último, hay que considerar que los profesionales de la salud, incluido el equipo de enfermería, se encuentran inmersos en un contexto formativo, asistencial, gerencial y de salud que puede imponer restricciones significativas a la atención de las necesidades específicas del niño y su familia. Así, cabe destacar que la práctica de contener al niño durante los procedimientos de salud parece verse afectada directamente por la formación y capacitación recibidas por los profesionales^(20)^. Estos son aspectos importantes que deben tenerse en cuenta en la reflexión y la fundamentación de los avances necesarios para garantizar los derechos de los niños y, en consecuencia, para que prevalezca una atención de enfermería basada en principios éticos.

Los resultados de este estudio están sujetos a limitaciones. Cabe destacar que los hallazgos aquí relatados se refieren a la experiencia de los profesionales de enfermería que trabajan en el sistema de salud de Brasil y, por lo tanto, se limitan a esa realidad asistencial. Además, hay que considerar que nuestro país se extiende por dimensiones continentales que pueden influir incluso en las prácticas de enfermería. Del mismo modo, cabe destacar que, a pesar de los esfuerzos de los investigadores, no se logró una muestra proporcional en la macrorregión Nordeste, en los estratos de enfermeros y técnicos/auxiliares de enfermería, y en la macrorregión Sur en el estrato de técnicos/auxiliares de enfermería, lo que puede interferir de alguna manera en las interpretaciones de los hallazgos. También deben tenerse en cuenta el tipo de muestra por conveniencia y el sesgo de memoria, ya que es posible que los profesionales de enfermería formados hace mucho tiempo no recuerden con precisión experiencias previas.

## CONCLUSIÓN

El método de contención se configuró como una práctica común para que el equipo de enfermería realizara procedimientos como punción venosa, administración de medicamentos y sondajes en los niños hospitalizados, siendo la contención física, la contención con apoyo y la contención psicológica las técnicas más empleadas. Su uso se justificó por el comportamiento del niño, la garantía de su seguridad, su negativa a cooperar y, además, por el grupo de edad. Además, los profesionales de enfermería priorizan a la familia y/o al responsable en detrimento del niño a la hora de dar explicaciones sobre la contención y obtener el consentimiento para llevarla a cabo. Tras la contención, era habitual ofrecer el regazo del familiar y refuerzo positivo para mitigar los posibles daños. En el expediente médico del niño, los profesionales registraban información sobre el procedimiento de salud y lo que motivó el uso de la contención, así como la presencia y el consentimiento de la familia y/o del responsable. Sin embargo, quedó evidenciado por el relato de los profesionales la falta de preparación del equipo de enfermería debido a la ausencia de contenidos sobre el tema en el proceso de formación y de actualización/capacitación durante la trayectoria profesional.

A la luz de los resultados, cabe destacar que estos pueden servir de base para la elaboración de directrices nacionales, la normatización profesional por parte del consejo de enfermería y el desarrollo de protocolos institucionales. Estos documentos deberán estipular recomendaciones para el equipo de enfermería sobre los aspectos relacionados con la práctica de la contención del niño hospitalizado y los dilemas éticos que se enfrentan en la atención clínica. Además, podrán fomentar la incorporación de este contenido y sus especificidades en el proceso de enseñanza y aprendizaje durante la formación en enfermería en el país, tanto a nivel técnico como en la licenciatura y la posgrado.

Por último, es fundamental destacar la necesidad de ampliar el alcance de este objeto de investigación en Brasil desde diferentes puntos de vista. Por ello, se insta a la realización de más investigaciones científicas que contemplen la contención en diversos contextos asistenciales, además de asegurar el protagonismo del niño. Esto permitirá que el fenómeno sea mapeado de manera integral, diversa y en sus más variadas peculiaridades, con el propósito de avanzar en las prácticas de enfermería con respeto y consideración hacia las emociones y los derechos de los niños.

## Data Availability

Todo el conjunto de datos que respalda los resultados de este estudio se publicó en el propio artículo.
